# A systematic scoping review of dissociation in borderline personality disorder and implications for research and clinical practice: Exploring the fog

**DOI:** 10.1177/00048674221077029

**Published:** 2022-02-13

**Authors:** Huda F Al-Shamali, Olga Winkler, Fernanda Talarico, Andrew J Greenshaw, Christine Forner, Yanbo Zhang, Eric Vermetten, Lisa Burback

**Affiliations:** 1 Department of Psychiatry, University of Alberta, Edmonton, AB, Canada; 2 Associated Counselling, Calgary, AB, Canada; 3 Department of Psychiatry, Leiden University Medical Center, Leiden, The Netherlands

**Keywords:** Borderline personality disorder, dissociation, dissociative disorder, self-harm, suicide

## Abstract

**Background:**

Borderline Personality Disorder (BPD) is frequently complicated by the presence of dissociative symptoms. Pathological dissociation is linked with earlier and more severe trauma exposure, emotional dysregulation and worse treatment outcomes in Posttraumatic Stress Disorder and Dissociative Disorders, with implications for BPD.

**Objective:**

A systematic scoping review was conducted to assess the extent of current literature regarding the impact of dissociation on BPD and to identify knowledge gaps.

**Methods:**

Four electronic databases (MEDLINE, APA PsycINFO, EMBASE, CINAHL Plus) were searched, and English peer-reviewed studies with adults with BPD were included, following Preferred Reporting Items for Systematic reviews and Meta-Analysis (PRISMA) extension for scoping reviews (PRISMA-ScR) 2018 guidelines.

**Results:**

Most of the 70 included studies were observational (98%) with first authors from Germany (59%). Overall, dissociation was associated with increased BPD symptom severity, self-harm and reduced psychotherapy treatment response; findings regarding suicide risk were mixed. Dissociation was associated with working memory and cognitive deficits, decreased pain perception, altered body ownership, no substance abuse or the abuse of sedative substances, increased fantasy proneness, personality fragmentation, fearful attachment, dream anxiety, perceived stress and altered stress responses, increased cumulative body mass index, decreased water consumption, several neurological correlates and changes in gene expression.

**Conclusion:**

BPD with significant dissociative symptoms may constitute a more severe and at-risk subgroup of BPD patients. However, there are significant research gaps and methodological issues in the area, including the possibility of unrecognized Dissociative Disorders in BPD study populations confounding results. Further studies are needed to better understand the impact of dissociation on BPD course and treatment, and to clarify the most appropriate assessment tools for clinical practice. In addition, interventional studies are needed to develop dissociation-specific BPD treatments to determine whether targeting dissociation in BPD can improve treatment outcomes.

## Introduction

### Borderline personality disorder

Once thought untreatable, great advances have been made in Borderline Personality Disorder (BPD) treatment, including evidence-based psychotherapies such as Dialectical Behavioral Therapy (DBT), Schema Focused Therapy (SFT) and Transference Focused Psychotherapy (TFP). However, these psychotherapies are expensive, access is poor and treatment often has limited impact on functional outcomes (Links et al., 2017). This is a significant problem in general psychiatry, given that BPD comprises 20% of psychiatric inpatients and carries a 10% risk of suicide (American Psychiatric Association, 2013). Dissociative symptoms are a criterion of BPD; features may include ‘*psychotic-like symptoms (e.g., hallucinations, body-image distortions, ideas of reference, hypnagogic phenomena) during times of stress*’ and ‘*self-mutilation may occur during dissociative experiences*’ (American Psychiatric Association, 2013). Up to 80% of BPD patients experience dissociative symptoms, and pathological dissociation has been linked to poor functional outcomes (Brand and Lanius, 2014; Korzekwa et al., 2009; Krause-Utz et al., 2017). Despite this, dissociative symptoms enjoy little emphasis in psychiatric training and clinical practice. However, understanding these phenomena may be a key element important to moving the treatment of BPD forward.

### Spectrum of dissociative phenomena

Dissociation is defined as a disruption of and/or discontinuity in the normal, subjective integration of consciousness, memory, identity, emotion, perception, body representation, motor control or behavior (American Psychiatric Association, 2013). Often occurring in response to intense emotions, psychological conflict or inescapable threat (Brand and Lanius, 2014), dissociative symptoms range from ordinary experiences of absorption to pathological dissociative states associated with distress and functional sequelae. Pathological dissociation can be categorized into primary, secondary and tertiary symptoms, which occur across a wide range of psychiatric diagnoses. Primary dissociation includes intrusive re-experiencing symptoms such as flashbacks, associated with emotional undermodulation, failure of corticolimbic inhibition and increased hyperarousal during memory activation. Secondary dissociation, including depersonalization and derealization, is associated with excess corticolimbic inhibition, reduced amygdala and insula activation, increased filtering of sensory information and hypoarousal. Tertiary dissociation includes development of identity states with distinct cognitive, affective and behavioral patterns and differential access to traumatic memories, which may present as internal voices or transient psychotic symptoms (Brand et al., 2012; Brand and Lanius, 2014; Loewenstein, 2018; Reinders et al., 2006). Secondary and tertiary dissociation are associated with illness severity and chronicity, higher treatment dropout, inhibited emotional learning and habituation, poor response to treatment, attachment difficulties and early, repeated or inescapable traumatic experiences (Brand and Lanius, 2014; Korzekwa et al., 2008, 2009; Krause-Utz and Elzinga, 2018; Loewenstein, 2018). Therefore, it follows that dissociation may also have implications for BPD (Krause-Utz et al., 2017).

Lack of emphasis on dissociation in BPD assessment may facilitate misdiagnosis, or underrecognition of important comorbidities. There is significant overlap between BPD and Dissociative Identity Disorder (DID); BPD is diagnosed in 30–70% of DID patients and Dissociative Disorders (DDs) are diagnosed in 41–72% of BPD patients. DDs may present with self-harm, suicidality, emotional dysregulation, intolerance to emotional experiencing and identity problems, and be misdiagnosed as BPD (see Brand and Lanius, 2014, for a review of chronic complex DDs and comparison between DDs and BPD). Posttraumatic Stress Disorder (PTSD) is comorbid with BPD in up to 56% of cases (Shah and Zanarini, 2018) and may also present with emotional dysregulation, reckless, aggressive or self-destructive behavior, identity disturbance or dissociative symptoms (American Psychiatric Association, 2013). Given the symptom overlap, assessing for a wide range of dissociative phenomena could improve diagnostic accuracy and recognition of important comorbidities requiring different treatment considerations.

### Measuring dissociation

Dissociation exists along a continuum, ranging from normal to pathological, and state to trait. State dissociation is a transient state linked to stress or traumatic cues (e.g. derealization during a traumatic event), whereas trait dissociation is a more enduring pattern (Krause- Utz et al., 2017). Existing dissociation scales vary in their sensitivity to detect state or trait dissociation and the range of dissociative phenomena measured. Common scales more sensitive for state dissociation include the Dissociative Tension Scale (DSS) and the Clinician Administered Dissociative States Scale (CADSS). The DSS is a 22-item self-report questionnaire with somatoform symptoms (i.e. altered pain perception, vision and hearing) and psychological dissociation (derealization, depersonalization, amnesia) (Stiglmayr et al., 2003); a shorter version (DSS-4) contains only four items (i.e. depersonalization, derealization, altered hearing and pain perception) (Stiglmayr et al., 2009). The CADSS is a 27-item scale sensitive to fluctuating dissociative symptoms over time (Bremner et al., 1998).

Scales sensitive to trait dissociation include the Dissociative Experiences Scale (DES), the Fragebogen zu Dissoziativen Symptomen (FDS) and the Multidimensional Inventory of Dissociation (MID). The DES, a 28-item self-rated scale, assesses tendency for derealization, depersonalization, dissociative amnesia, absorption (imaginary involvement) and identity disturbances, becoming more sensitive to trait dissociation with increasing scores; DES scores above 30 increasingly signal higher probability of a DD (Carlson and Putnam, 1993). The FDS is the 44-item German version of the DES, which includes additional somatic and conversion symptom questions (Spitzer et al., 1998). The MID, a 218-item self-report questionnaire, provides a comprehensive assessment of dissociative symptoms, contains validity scales and reports a probability for specific diagnoses, including BPD, DID and PTSD (Dell, 2006).

### Objectives

Given the high prevalence and potential clinical implications, there is a need to understand the consequences of dissociation for the course and treatment of BPD. Given the dearth of interventional trials and the heterogeneous literature, a scoping review approach was used to map the current state of evidence and identify knowledge gaps for future research. The objectives included to (a) systematically search the literature for studies on the impact of dissociation in BPD, and (b) report on the range of outcomes associated with dissociation in BPD populations. In addition, because of heterogeneity of BPD populations, the high rate of comorbidity and the potential for dissociation scales such as the DES to imply potential comorbidities, the dissociation scales used and sample means were also reported. Given the impact of medications on dissociative symptoms, medication status of study populations was also included.

## Methods

This scoping review followed the Preferred Reporting Items for Systematic reviews and Meta-Analysis extension for scoping reviews (PRISMA-ScR) 2018 guidelines (Tricco et al., 2018). The review protocol was created a priori but was not registered.

### Information sources

On May 6, 2021, a systematic search of the following databases was conducted: MEDLINE (Ovid Interface 1946–2021), APA PsycINFO (Ovid Interface 1806–2021), EMBASE (Ovid Interface 1974–2021) and CINAHL Plus with Full Text (EBSCOhost Interface 1937–2021). The employed search strategy used a mix of keyword synonyms and subject headings for two main topics: borderline personality disorder and dissociation. In addition, the bibliographies of pertinent reviews were searched for relevant publications. Full details of the search strategy are available in Supplementary Materials 1.

### Eligibility criteria

Articles were included if they reported on any effect that was correlated with or attributed to the presence of dissociative symptoms in BPD patients. Only adults aged 18 years and older who were diagnosed with BPD according to Diagnostic and Statistical Manual of Mental Disorders (*DSM*) or the International Classification of Diseases (ICD) criteria were included. For reasons related to feasibility and cost, this review included peer reviewed qualitative and quantitative articles written in English. Review papers and books were excluded since they are a collection of information and studies likely already included as part of our search. Unpublished dissertations were excluded as we adopted the formal peer review standard associated with peer-reviewed journals as a significant criterion for information quality.

### Data screening and extraction

Articles retrieved by the search strategy were uploaded into the Covidence program (covidence.org) where duplicates were automatically removed. Two independent reviewers screened the articles over two phases: abstract/title and full-text screening. Discrepancies were resolved through discussion and consensus. After screening was completed, data were extracted by one reviewer independently and reviewed by a second reviewer. The data extraction form was developed through team discussion and created using Google Form. Extracted information included country of first author, study design, instrument used for BPD diagnosis, sample size, mean age and standard deviation, sex and gender, participant characteristics, dissociation measurement instrument, outcomes associated with dissociation in BPD and measures or tests used to evaluate the impact of dissociation. Participant characteristics included diagnosis attributed to the dissociative symptoms (symptom of BPD, DD or comorbid PTSD), whether participants were inpatients, outpatients or not reported (N/R) and participant medication status (medicated, unmedicated or N/R).

### Data synthesis

A thematic qualitative analysis was conducted, and articles were evaluated based on reported effects of dissociative symptoms in patients with BPD (Levac et al., 2010). Articles that discussed similar effects of dissociation were collated and analyzed together, as described in the results section and as presented in the supplementary tables.

## Results

**Figure 1.**
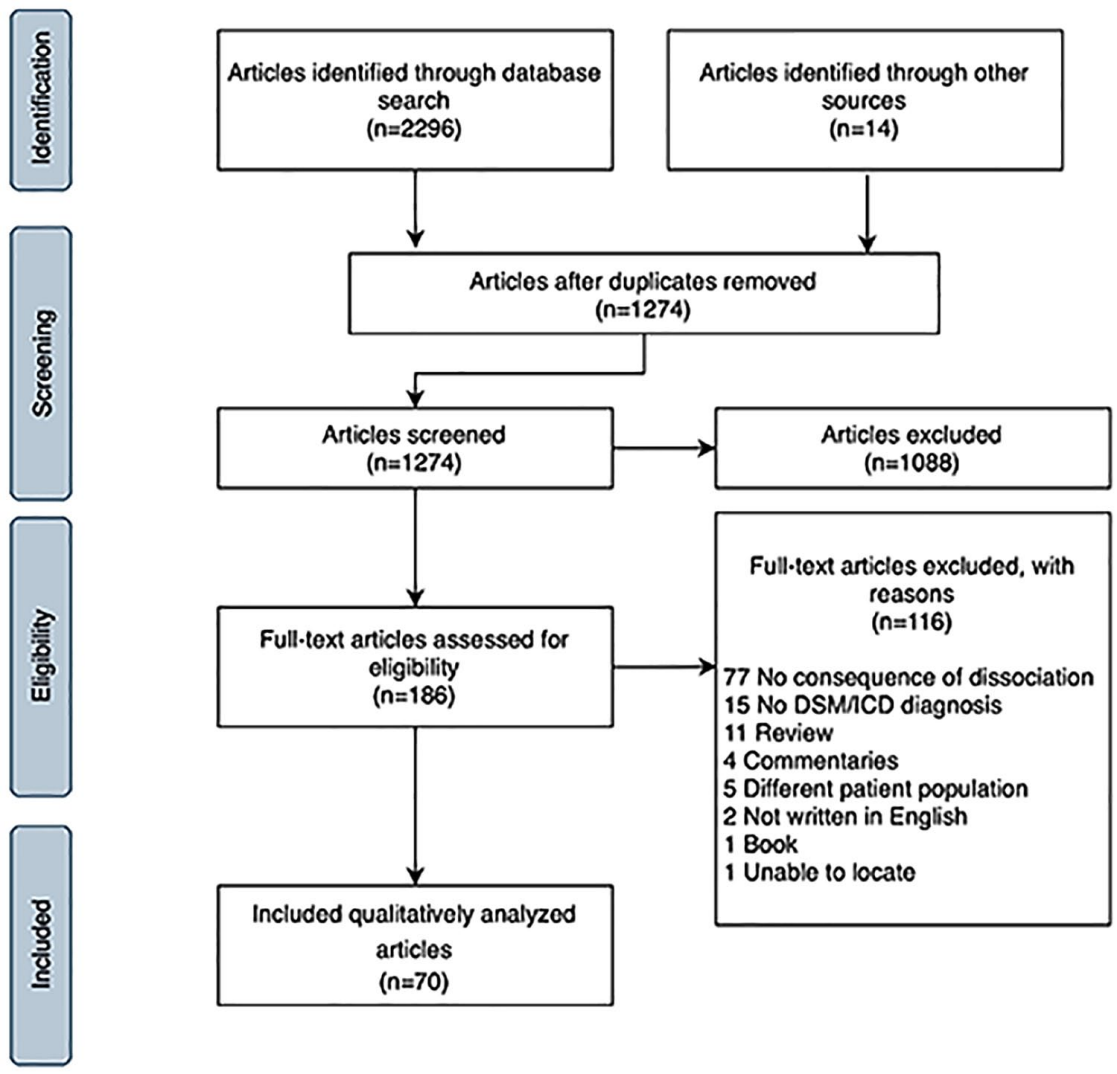
PRISMA flow diagram.

The database search identified 2296 papers and 14 were added from reference lists of relevant reviews. After removal of duplicates, 1274 abstracts were screened, and 1088 excluded. Of the remaining 186 full-text studies assessed for eligibility, 116 were excluded, leaving 70 articles for analysis (Figure 1).

### Study and sample characteristics

The first authors of the 70 included articles were from 12 countries, mostly Germany (41 studies; 59%), followed by the United States (14 studies; 20%). The earliest study was published in 1993, and 54% were published after 2009. All included articles were observational studies, except for one randomized control trial (Table 1).

**Table 1.** Study characteristics.

Study characteristics	Number (%) of references (*n* = 70)
Country of publication	
Germany	41 (58.6%)
United States of America	14 (20.0%)
Other European countries	13 (18.6%)
Canada	1 (1.4%)
Israel	1 (1.4%)
Year of publication	
1991–2000^a^	9 (12.9%)
2001–2010	26 (37.1%)
2011–2021	35 (50%)
Publication design	
Randomized control trial	1 (1.4%)
Cross-sectional study	59 (84.3%)
Longitudinal study	7 (10.0%)
Retrospective study	2 (2.9%)
Case-control study	1 (1.4%)

a First paper was published in 1993.

The studies included 4818 participants, 90% of whom were female. Of the studies reporting on gender, one study used transsexuality as an exclusion criteria, and one study mentioned two participants with gender identity disorder. The greater proportion of studies enrolled inpatients (26 studies; 37%), medicated patients (32 studies; 46%) and participants diagnosed using the *DSM* (69 studies; 99%) (Table 2 Sample Characteristics). The most utilized rating scales were the DES or its expanded German version, the FDS (55 studies; 78.6%), followed by the DSS and its shorter version the DSS-4 (23 studies, 32.9%) (see Table 3).

**Table 2.** Sample characteristics.

Sample characteristics	Number (%) of references (*n* = 70)
Patient type	
Inpatient	26 (37.1%)
Outpatient	16 (22.9%)
Both	17 (24.3%)
Not reported	11 (15.7%)
Medication status	
Medicated	32 (45.7%)
Unmedicated	25 (35.7%)
Not reported	13 (18.6%)
BPD diagnosis	
*DSM*	68 (97.1%)
ICD	1 (1.4%)
*DSM* and ICD	1 (1.4%)

*DSM*: Diagnostic and Statistical Manual of Mental Disorders; BPD: Borderline Personality Disorder; ICD: International Classification of Diseases.

**Table 3.** Correlations and measurements of dissociation.

	Number (%) of references (*n* = 70)
Correlations of dissociation	
Neural correlates	17 (24.3%)
Pain thresholds	15 (21.4%)
Physiological correlates	14 (20.0%)
Self-harm	11 (15.7%)
Psychological correlates	10 (14.3%)
Memory	8 (11.4%)
Behavioral correlates	4 (5.7%)
Psychotherapeutic treatment	3 (4.3%)
Emotional learning	3 (4.3%)
BPD symptom severity	2 (2.9%)
Suicide	2 (2.9%)
Gene expression	1 (1.4%)
Measures of dissociation	
DES/FDS	55 (78.6%)
DSS/DSS-4	23 (32.9%)
SCID-D	4 (5.7%)
DES-Taxon	2 (2.9%)
WDS	1 (1.4%)
PDEQ	1 (1.4%)
BPDSI-IV	1 (1.4%)
SCID-II	1 (1.4%)
*DSM*-II	1 (1.4%)
4-Questions (DES and SDQ-5)	1 (1.4%)

BPDSI-IV: Borderline Personality Disorder Severity Index–Version Four; DES-Taxon: Dissociative Experiences Scale–Taxon; DES: Dissociative Experiences Scale; *DSM*-II, Diagnostic and Statistical Manual of Mental Disorder–Second Edition; DSS-4: Dissociative Tension Scale–4 Items; DSS: Dissociative Tension Scale; FDS: Fragebogen zu Dissoziativen Symptomen; PDEQ: Peritraumatic Dissociative Experience Scale; SCID-D: Structured Clinical Interview for the *DSM-IV* Dissociative Disorders; SCID-II: Structured Clinical Interview for the *DSM-IV* Axis II Personality Disorders; SDQ-5: Somatoform Dissociation Questionnaire; WDS: Wessex Dissociation Scale.

### Overview of findings related to impact of dissociation

Approximately 50 correlates of dissociation were reported, including neural correlates or neuroimaging (17 studies; 24%), pain thresholds or perception (15 studies; 21%), physiological correlates (14 studies; 20%), self-harm (11 studies; 16%), psychological outcomes (10 studies; 14%), memory (eight studies; 11%), behavioral correlates (four studies; 6%), emotional learning (three studies; 4%), treatment impact (three studies; 4%), symptom severity (two studies; 3%), suicide risk (two studies; 3%) and gene expression (one study; 1%) (see Table 3). Study characteristics, study objectives, main dissociation-related outcome, intervention, dissociation scale scores and main results are outlined in Supplementary Materials 2.

### BPD and associated symptoms

Dissociation was associated with greater severity of BPD and associated symptoms, including increased depressive symptoms, anxiety, perceived stress, behavioral dyscontrol, hopelessness, self-harm, alcohol abuse, PTSD symptoms and more frequent hospital admissions (Demirkol et al., 2020; Hoerst et al., 2010; Jaeger et al., 2017; Miller et al., 1993; Shearer, 1994). Derealization and depersonalization were correlated with both the absence of substance abuse, and increased alcohol or sedative use, but rarely stimulants (Miller et al., 1993). Similarly, Shearer (1994) reported that higher DES scores (mean DES = 25.02 [SD = 21.19]) were positively correlated with alcohol abuse.

Dissociation was also associated with brain structures and paradigms related to self-image and sense of self. Irle et al. (2007) observed a larger left postcentral gyrus in participants with BPD and comorbid DID or dissociative amnesia, and a correlation between symptoms of derealization and a larger right precuneus (associated with identity disturbances, altered self-evaluation and introspection). Moreover, higher FDS scores correlated negatively with subjective body ownership (Löffler et al., 2020) and DSS-4 and FDS scores correlated with increased sense of body plasticity (Bekrater-Bodmann et al., 2016), which refers to individuals’ ability to adopt an object within the peripersonal space as their own. Finally, decreased resting metabolic activity in temporoparietal areas, posterior cingulate cortex (PCC) and the left precuneus, areas associated with disturbed body perception, correlated with increased DES scores (Lange et al., 2005).

Recurrent self-harming behavior was generally positively associated with dissociative symptoms. Eight studies observed an association between dissociation and self-harm, using questionnaires more sensitive to trait dissociation than state dissociation (Brodsky et al., 1995; Colle et al., 2020; Kemperman et al., 1997; Kleindienst et al., 2008; Ludäscher et al., 2009; Navarro-Haro et al., 2015; Shearer, 1994; Zanarini et al., 2011); one study also used a state-sensitive measure (Navarro-Haro et al., 2015). Two studies found dissociation peaked during self-harm and significantly decreased after self-harm was complete (Kemperman et al., 1997; Kleindienst et al., 2008). In addition, participants with BPD and higher DES scores experienced less pain during self-harm (Kemperman et al., 1997). Kleindienst et al. (2008) reported that self-harm was primarily used to reduce aversive tension (51% of patients), reduce unpleasant emotions (13%), or as a form of self-punishment (12%). However, some participants reported engaging in self-harm to reduce dissociative symptoms (8% to recover control; 7% to regain awareness of physical sensations; 7% to regain a sense of reality) (Kleindienst et al., 2008).

Two studies assessed the correlation of dissociation with suicide risk, with conflicting results (McGirr et al., 2009; Wedig et al., 2012). McGirr et al. (2009) completed a retrospective observational study using proxy-based interviews of family and friends of a mainly male BPD population who died by suicide, finding a negative correlation between dissociative symptoms and suicide risk (McGirr et al., 2009). Conversely, Wedig et al. (2012) conducted a 16-year longitudinal study that followed a group of 264 mostly female BPD inpatients; severe dissociation correlated positively with risk of suicide attempts (mean baseline DES score = 21.8 [SD = 18.6]) (Wedig et al., 2012).

### Other psychological correlates

Higher scores on dissociative scales (DES or the Wessex Dissociation Scale) correlated with multiple psychological sequelae, including fantasy proneness (Merckelbach et al., 2005), increased personality division, characterized by ‘*schema modes*’ (Johnston et al., 2009), and fearful attachment (Simeon et al., 2003). Finally, higher DES scores in BPD with comorbid nightmare disorder positively correlated with dream anxiety (Semiz et al., 2008).

### Memory, cognition, and emotional learning

Several studies investigated associations between dissociative symptoms and memory, cognition and emotional learning. There were mixed results regarding specificity of autobiographical memories (Jones et al., 1999; Kremers et al., 2004; Renneberg et al., 2005). High DES scores (mean DES 39.9[SD = 17]) correlated negatively with specificity of recalled autobiographical memory when compared with healthy controls (DES 8.9[SD = 7.3]) (Jones et al., 1999); other studies found no correlation, although DES scores were lower in these studies (mean DES score = 23.4–23.9[SD = 14–11.8]) (Kremers et al., 2004; Renneberg et al., 2005). Three studies found impaired working memory in patients with BPD (Krause-Utz et al., 2014a, 2018; Stevens et al., 2004). Two of these studies observed a negative correlation between working memory and state dissociation, measured by the DSS-4, which was hypothesized to be related to greater emotional distraction (Krause-Utz et al., 2014a, 2018).

The association between emotional distraction or emotional learning and dissociation was assessed by five studies, also with mixed results. Two studies employed an Emotional Stroop Task (EST) and observed longer reaction times and inefficient cognitive inhibition in patients with BPD (Wingenfeld et al., 2009; Winter et al., 2015). However, only one of studies observed a correlation with increased DSS scores (Winter et al., 2015). Emotional distraction, brought on by dissociation, may also impair emotional learning in patients with BPD and significant state dissociation (high DSS scores) (Ebner-Priemer et al., 2009; Paret et al., 2016). However, another study did not observe a correlation between trait dissociation (DES scores) and impaired emotional learning (Krause-Utz et al., 2015).

In addition to studies on emotional learning and working memory, Haaland and Landrø (2009) reported that, compared with healthy controls, BPD patients with higher DES scores performed worse across multiple cognitive domains (executive functioning, attention, long-term verbal memory, working memory and general cognition), whereas BPD patients with lower DES scores only exhibited decreased executive functioning.

### Neurological correlates

Nine studies reported on neurological correlates impacted by dissociation (Irle et al., 2005; Krause-Utz et al., 2014a, 2014b, 2018; Niedtfeld et al., 2013; Popkirov et al., 2018; Prossin et al., 2010; Rusch et al., 2007; Wolf et al., 2012). Findings within this set of studies include a correlation between higher FDS or DES scores and brain gray matter volume in the middle temporal gyrus (Niedtfeld et al., 2013), mean diffusivity in the right inferior frontal white matter (Rusch et al., 2007), and increased non-displaceable binding potential (BPND), corresponding to decreased levels of opioid regulatory control and increased sensitivity to emotional stimuli (this correlation did not remain significant after correction for multiple comparisons) (Prossin et al., 2010). In addition, a resting state functional magnetic resonance imaging (fMRI) study reported trait dissociation (DES) correlated with increased connectivity between the amygdala and dorsolateral prefrontal cortex, and negatively with functional connectivity between the amygdala and the cuneus, occipital lobe (V1), and fusiform gyrus (Krause-Utz et al., 2014b). These changes in resting state functional connectivity are proposed to underlie alterations in individual self-referential processes and negative emotion processing (Krause-Utz et al., 2014b, 2018). Finally, Popkirov et al. (2018) observed baseline frontal electroencephalogram (EEG) leftward asymmetry in BPD patients, compared with healthy controls, which positively correlated with FDS scores. Two studies reported neurological findings in BPD which were not associated with dissociation. Wolf et al. (2012) observed altered blood flow to the orbitofrontal cortex and Irle et al. (2005) reported that BPD was associated with a smaller right parietal cortex and leftward parietal asymmetry; neither was correlated with measures of dissociation.

### Pain threshold and pain perception

Dissociation in BPD was associated with decreased pain perception in 11 studies, using techniques to assess pain perception via thermal or electrical stimulation (Bekrater-Bodmann et al., 2015; Bohus et al., 2000; Cárdenas-Morales et al., 2011; Chung et al., 2020; Defrin et al., 2019; Kluetsch et al., 2012; Ludäscher et al., 2007, 2010; Russ et al., 1993, 1996, 1999), with five studies excluding patients taking psychotropic medication and five enrolling medicated patients. Medications included selective serotonin reuptake inhibitors (SSRIs), clomipramine, benzodiazepines, typical antipsychotics and the mood stabilizers lithium and lamotrigine. Five cross-sectional studies also examined the association between dissociation and pain thresholds in patients with BPD, with mixed results (Bekrater-Bodmann et al., 2015; Cárdenas-Morales et al., 2011; Chung et al., 2020; Defrin et al., 2019; Ludäscher et al., 2007).

Pain perception and sensitivity were associated with the posterior default mode network (DMN). Kluetsch et al. (2012) found that activity in the posterior DMN was negatively correlated with trait dissociation (FDS scale) in the BPD patient group. Results indicated that patients who experience more dissociative symptoms may perceive pain as less salient due to this change in brain activity (Kluetsch et al., 2012). In addition, Wolf et al. (2011) found that increased dissociation (mean DSS score = 38.8 [SD = 28.5]) positively correlated with abnormal connectivity in the insula of the DMN and negatively correlated with connectivity of the cuneus. The researchers reported that the observed change in insula activity was associated with a reduction in pain perception (Wolf et al., 2011). Pain sensitivity was found to be similar in BPD compared with BPD with comorbid PTSD (BPD + PTSD), but the BPD + PTSD group showed more prominent amygdala deactivation during painful stimulation; dissociation was not correlated with these amygdala changes (Kraus et al., 2009).

### Physiological correlates

Dissociation was associated with alterations in stress response and other physiologic parameters. Dissociation correlated with decreased heart rate during emotional memory processing (Bichescu-Burian et al., 2017) and emotional regulation tasks (Krause- Utz et al., 2019), lower amygdala activity when viewing emotional pictures (Hazlett et al., 2012; Krause-Utz et al., 2012), and reduced startle response (Barnow et al., 2012; Ebner-Priemer et al., 2005). Hypothalamic-pituitary-adrenal (HPA) axis dysregulation and increased cortisol levels were also associated with higher scores on the DES (Simeon et al., 2007) and the DSS (Fernando et al., 2012). Both papers provide evidence that dissociation may be positively correlated with biological vulnerabilities to stress. Other physiological correlates associated with increased FDS or DES scores are reduced water consumption (Hoeschel et al., 2008) and increased cumulative body mass index (cBMI) (Frankenburg and Zanarini, 2011).

### Gene expression

One study attempted to measure the impact of dissociative states on gene expression. Schmahl et al. (2013) reported that higher DSS scores positively correlated with interleukin-6 gene expression, and negatively correlated with the expression of other genetic markers related to immune system activation, intercellular signaling and HPA axis regulation.

### Response to psychotherapy treatment

Treatment effectiveness may be diminished for patients with BPD in the presence of dissociative symptoms (Arntz et al., 2015; Kleindienst et al., 2011; Pec et al., 2021; Spinhoven et al., 2006). Higher DES scores were correlated with less improvement during a 3-month DBT treatment (Kleindienst et al., 2011) and a decrease in both mindfulness and acting with awareness (Didonna et al., 2019). Similarly, Arntz et al. (2015) reported that dissociative symptoms may have a detrimental impact on the probability of recovery from BPD in outpatients treated with Schema Therapy (ST) or Transference Focused Psychotherapy (TFP). Assessed with the BPD checklist, 73% and 47% of participants with the lowest levels of dissociation responded to ST and TFP, respectively. Response rates dropped to 32% (ST) and 14% (TFP) for patients with the highest levels of dissociation (Arntz et al., 2015). Conversely, Pec et al. (2021) did not observe DES scores to be a negative predictor of treatment outcome for participants in a 9-month group psychodynamic day treatment program. However, there was a positive correlation between DES scores and treatment dropout rates.

## Discussion

The peer reviewed literature on dissociation in BPD indicates that dissociation is associated with many important clinical, psychological and biological parameters. Clinically important correlations include BPD severity, self-injurious behavior, decreased psychotherapy treatment response, altered patterns of substance misuse, altered stress physiology and pain perception, working memory and cognitive deficits, fantasy proneness, personality fragmentation and fearful attachment.

Despite the growing literature on dissociation in BPD, many important methodological issues and research gaps remain (see Table 4). In particular, there is a paucity of randomized controlled trials, including studies testing potential treatments to address dissociation. Because almost all studies were observational, causality cannot be inferred from these data. Generalizability is impacted by the lack of male participants and limited studies outside of Germany and the United States. There is also a dearth of information about dissociation in relation to gender identity outside a binary lens, which is important given the association of dissociation with alterations in self-perception and identity (Keating and Muller, 2020). In addition, almost half the studies included medicated patients, which may be a confounding factor.

**Table 4.** Key knowledge gaps and research questions for future studies in BPD.

Subject	Knowledge gap	Research questions
Causality	98% of studies are observational (causality not investigated)	What is the nature of the relationship between dissociation and associated BPD outcomes (BPD severity, psychotherapy response, etc.)?
Dissociation type	Studies did not distinguish between dissociation subtypes phenomenologically	Do primary, secondary and tertiary dissociation subtypes have different impacts on BPD outcomes? Is state or trait dissociation more important to treatment efficacy? Are there specific dissociative phenomena that impact outcomes more than others (e.g., hostile dissociative voices, dissociative amnesia)?
Impact of assessing dissociation in BPD	Limited attention on dissociation assessment for BPD in clinical practice	Can assessment of dissociation lead to useful information about treatment outcomes, risk factors for self-harm or suicidality, or need for particular treatments? Does tracking dissociative symptoms provide useful clinical information? What measures are most appropriate?
BPD diagnosis	Significant overlap between BPD, PTSD and DDs. Studies often do not account for possible comorbidities	Can assessment measures accurately differentiate between DDs, PTSD and BPD?
Substance abuse	Association between dissociation and sedative substance	Can this finding be replicated? What are the potential mechanisms? Can treatment of dissociation reduce sedative or alcohol misuse?
Sex, gender and gender identity	No studies addressing the role of gender or gender identity 90% of participants female	Are certain dissociative symptom subtypes more prevalent in certain sexes or genders? Is there a relationship between dissociation severity and gender identity? Are there sex or gender differences in the outcomes associated with dissociation?
Demographics	80% of studies were in Germany or USA	Are there differences in how dissociation presents in BPD populations across cultures?
Self-harm	Some BPD patients use self-harm to manage dissociation	Do certain subtypes of dissociation, or dissociation severity, have different implications for self-harm?
Suicidality	Few studies; mixed results	Do dissociative symptoms impact suicidality? Do specific subtypes of dissociative symptoms have different impacts on suicidality?
Cognitive function	Dissociation is associated with a range of memory and cognitive deficits	Are the deficits caused by dissociation? Which BPD patients are vulnerable to dissociation impacting cognitive assessments?
Psychotherapy	Dissociative symptoms are associated with poorer psychotherapy outcomes	Would dissociation specific treatments improve BPD outcomes?
Pharmacology	Research indicates reduced pain perception and altered threshold, and potential role of the opioid system	Would naloxone or other opioid antagonists improve dissociation related outcomes in BPD?
Physiologic state	Dissociation associated with increased cBMI and reduced water intake	What is the mechanism of these findings? Is dissociation associated with alterations of other drives, such as psychomotor activity, sexual behaviors, or maternal behaviors in BPD?

BPD: Borderline Personality Disorder; PTSD: Posttraumatic Stress Disorder.

Interpretation of results is also hampered by the lack of precise and consistently applied definitions for dissociative phenomena and the absence of a clinically oriented assessment tool capable of guiding diagnosis and treatment. The majority of studies used the DES, FDS, or DSS, none of which provide diagnostic clarity. Furthermore, dissociation includes a wide variety of phenomena ranging from transient experiences of disconnection to complex altered states of consciousness. Most studies referred to dissociation in the context of scores on scales without reporting on clinical context or associated phenomenology. It is possible that subgroups of dissociative phenomena have different neurobiological features and functional consequences, requiring different strategies. Research regarding the range, neurobiology and relative impact of different types of dissociation in BPD are needed.

Adding to the confusion, BPD studies often did not explicitly report whether common confounding conditions were identified or excluded. Only eight studies mentioned whether the sample population included those with DDs, and only two explicitly excluded DID. Forty-three reported inclusion of participants with comorbid PTSD, while eight excluded them. DDs are often underrecognized and, because DD patients may present with self-harm, suicidality, emotional dysregulation and identity problems, DDs can be misdiagnosed as BPD (Brand and Lanius, 2014). Previous research reports DES score ranges of 18–20 for BPD, 26–41 for PTSD and 43–57 for DID (Carlson and Putnam, 1993). In the BPD studies included in this review, mean DES scores for BPD groups ranged from 10.5 [SD = 7.2] (Haaland and Landrø, 2009) to 52.4 [SD = 15.8] (Semiz et al., 2008), indicating significant heterogeneity across samples. No studies used the MID, which provides a probability score for the presence of various diagnoses, such as BPD, DID and PTSD (Dell, 2006). Therefore, it is possible that some of the findings related to dissociation in BPD were actually a consequence of DDs, PTSD, or a combination, rather than BPD itself. This may account for the mixed results in studies investigating impacts on working memory, cognition and emotional learning, as study groups had wide variability in mean dissociation scores. This highlights the need to develop clinically appropriate assessment tools for dissociation in BPD to reduce misdiagnosis, assess for dissociation related treatment targets and to track treatment progress.

Despite these challenges, the current literature signals that dissociation is a potential marker for both BPD severity and poor treatment response, indicating a need to better understand the mechanisms involved and develop strategies to address them. In the PTSD literature, dissociative symptoms are linked to clinical severity and lower response to psychotherapy (Kim et al., 2019; Lanius et al., 2012). This reduced response has been explained by data demonstrating prefrontal inhibition of limbic regions, leading to emotional overmodulation in response to emotional or traumatic cues (Lanius et al., 2010; Lyssenko et al., 2018). One clinical implication is that such patients may have difficulty responding to trauma therapy due to both impaired emotional regulation capacities and a tendency to dissociate on exposure to distressing cues inherent in processing the trauma (Lanius et al., 2010). This parallels results found in this BPD review, pointing to a dysfunctional HPA axis and high stress responsiveness, with suppressed amygdala activity, increased emotional distraction and a variety of dissociation-related attentional and cognitive deficits in BPD participants with dissociation. Clinically, dissociation was associated with poorer response to ST, TFP and a 3-month DBT program (Arntz et al., 2015; Kleindienst et al., 2011; Spinhoven et al., 2006). Additional research is needed to further understand the relative impact of both trait dissociation and in-session state dissociation on current treatments, as well as the potential for pre-treatment with specific interventions to address dissociation before entering standard, resource intensive psychotherapies for BPD.

The relationship between dissociation and risk of suicidal and parasuicidal behaviors is another important area requiring further inquiry. Only two studies focused on dissociation and suicide risk, and methodological issues preclude drawing any conclusions. McGirr et al. (2009) used retrospective, post-mortem reports of dissociation symptoms by family members, which was likely impacted by both recall bias and limited knowledge of the deceased person’s subjective experiences. In addition, dissociation was measured using the Structured Clinical Interview for the *DSM-IV* Axis II Personality Disorders (SCID-II) BPD-related dissociation questions, likely limiting the range of dissociation symptoms captured. In contrast, Wedig et al. (2012) prospectively collected data directly from BPD patients using the DES. However, the study population had relatively low DES scores (mean = 21.8[SD = 18.6]), which may have accounted for the low odds ratio (OR) as a predictor for suicide attempts (OR: 1.02, *p* = 0.002). Elucidating any important subgroups may be important in future studies, as it is possible that the relationship is complex and may depend on the type, severity and complexity of the dissociative phenomena (internal derogatory voices, amnesia, alter personality states, etc).

The relationship between dissociation and non-suicidal self-injury (NSSI) may also be complex. Eight studies examined this relationship, overall finding a positive correlation. Studies focused on measures of trait dissociation, rather than measuring state dissociation, which may have resulted in underestimation of the impact of dissociative processes during NSSI (Brand et al., 2009). Both dissociation and NSSI have been associated with opioid release and altered pain perception, potentially implicating the opioid system (Lanius et al., 2018). Kleindienst et al. (2008) reported BPD participants who did not feel pain during NSSI scored very high on the DES (mean = 43.6 [SD = 19.2]) compared with a group who self-harmed but felt pain (mean DES = 25.8[SD = 16.2]). It would be useful to know if the group differences correlated with differences in subjective experiences such as the presence of derogatory or command dissociative voices, amnesia for self-harm episodes, or altered sense of agency just before or during the self-harm episode. These phenomena have been associated with higher DES scores, typically above 40, and greater personality fragmentation (Carlson and Putnam, 1993; Johnston et al., 2009). Clinically, patients report a range of subjective experiences related to NSSI, such as self-harm urges arising from overwhelming tension or painful emotions, self-harm to reduce numbing, derealization, depersonalization or internal voices, self-harm during amnestic episodes and/or self-harm experienced as happening without agency in the context of dissociation (Kleindienst et al., 2008). Each of these may represent a different subgroup, with specific associated neurobiology, dissociative phenomenology and effective treatment approaches.

## Limitations

This review systematically searched articles from four electronic databases, but only articles written in English were included, limiting the scope of the review. In addition, dissertations and gray literature were excluded, applying a quality standard of indexed journal peer review. Therefore, we may have missed some findings yet to be published.

## Conclusion

Despite research indicating that pathological dissociation is associated with misdiagnosis, clinical severity, comorbidity and poor treatment outcomes, little attention has been paid to dissociation in BPD research and clinical practice, even though dissociation is experienced by up to 80% of BPD patients. This scoping review found dissociation in BPD to be correlated with BPD symptom severity, self-harm, poor psychotherapy treatment response, reduced mindfulness, sedative substance abuse, cognitive deficits, personality fragmentation, fearful attachment, altered pain perception, altered stress response and multiple other psychological, neurological and physiological correlates. This indicates that those with significant dissociative symptoms may constitute a more severe and at-risk subgroup of BPD patients, who may require different treatment strategies. However, there are significant research gaps and methodological issues in the area, including the possibility of unrecognized Dissociative Disorders in BPD study populations confounding results. Further studies are needed to better understand the impact of dissociation on BPD course and treatment, and to clarify the most appropriate assessment tools for clinical practice. In addition, interventional studies are needed to develop dissociation-specific BPD treatments to determine whether targeting dissociation in BPD can improve treatment outcomes.

## Supplemental Material

sj-docx-1-anp-10.1177_00048674221077029 – Supplemental material for A systematic scoping review of dissociation in borderline personality disorder and implications for research and clinical practice: Exploring the fog Click here for additional data file. Supplemental material, sj-docx-1-anp-10.1177_00048674221077029 for A systematic scoping review of dissociation in borderline personality disorder and implications for research and clinical practice: Exploring the fog by Huda F Al-Shamali, Olga Winkler, Fernanda Talarico, Andrew J Greenshaw, Christine Forner, Yanbo Zhang, Eric Vermetten and Lisa Burback in Australian & New Zealand Journal of Psychiatry

sj-docx-2-anp-10.1177_00048674221077029 – Supplemental material for A systematic scoping review of dissociation in borderline personality disorder and implications for research and clinical practice: Exploring the fog Click here for additional data file. Supplemental material, sj-docx-2-anp-10.1177_00048674221077029 for A systematic scoping review of dissociation in borderline personality disorder and implications for research and clinical practice: Exploring the fog by Huda F Al-Shamali, Olga Winkler, Fernanda Talarico, Andrew J Greenshaw, Christine Forner, Yanbo Zhang, Eric Vermetten and Lisa Burback in Australian & New Zealand Journal of Psychiatry
