# Supplementary material for: A systematic scoping review of dissociation in borderline personality disorder and implications for research and clinical practice: Exploring the fog
Source: Aust N Z J Psychiatry. 2022 Feb 13;56(10):1252–64. doi: 10.1177/00048674221077029 (PMC9511244; doi:10.1177/00048674221077029)
Supplement: sj-docx-1-anp-10.1177_00048674221077029 – Supplemental material for A systematic scoping review of dissociation in borderline personality disorder and implications for research and clinical practice: Exploring the fog [file sj-docx-1-anp-10.1177_00048674221077029.docx]

**Supplementary Material 1: Search Strategy**

**Ovid MEDLINE(R) ALL 1946 to May 5, 2021**

Date searched: May 6, 2021

Results= 544

1. Dissociat*.mp.
2. Exp Dissociative disorders/
3. (Depersonali* or dereali*).mp.
4. Depersonalization/
5. 1 or 2 or 3 or 4
6. Borderline Personality Disorder/
7. (Borderline personality disorder or BPD).mp.
8. (Emotionally unstable personality disorder or EUPD).mp.
9. 6 or 7 or 8
10. 5 and 9

**APA PsycInfo 1806 to May Week 1 2021**

Date searched: May 6, 2021

Results=880

1. Dissociat*.mp.
2. Exp Dissociative disorders/
3. (Depersonali* or dereali*).mp.
4. Depersonalization/
5. 1 or 2 or 3 or 4
6. Borderline Personality Disorder/
7. (Borderline personality disorder or BPD).mp.
8. (Emotionally unstable personality disorder or EUPD).mp.
9. 6 or 7 or 8
10. 5 and 9

**Embase 1974 to 2021 May 5**

Date searched: May 6, 2021

Results= 697

1. Dissociat*.mp.
2. Exp Dissociative disorder/
3. (Depersonali* or dereali*).mp.
4. Depersonalization/
5. 1 or 2 or 3 or 4
6. Borderline Personality Disorder/
7. (Borderline personality disorder or BPD).mp.
8. (Emotionally unstable personality disorder or EUPD).mp.
9. 6 or 7 or 8
10. 5 and 9
11. Limit 10 to conference abstracts
12. 10 not 11

**CINAHL full text 1937**

Date searched: May 6, 2021

Results= 175

1. (MH "Dissociative Disorders+")
2. dissociat* or Depersonali* or dereali*
3. (MH "Depersonalization")
4. S1 OR S2 OR S3
5. (MH "Borderline Personality Disorder")
6. borderline personality disorder or bpd or emotionally unstable personality disorder or eupd
7. S5 OR S6
8. S4 AND S7
