# Supplementary material for: A systematic scoping review of dissociation in borderline personality disorder and implications for research and clinical practice: Exploring the fog
Source: Aust N Z J Psychiatry. 2022 Feb 13;56(10):1252–64. doi: 10.1177/00048674221077029 (PMC9511244; doi:10.1177/00048674221077029)
Supplement: sj-docx-2-anp-10.1177_00048674221077029 – Supplemental material for A systematic scoping review of dissociation in borderline personality disorder and implications for research and clinical practice: Exploring the fog [file sj-docx-2-anp-10.1177_00048674221077029.docx]

**Supplementary Material 2**

**Table 1a:** Study characteristics and main findings.

| **Study and country** | **Design** | **BPD Diagnosis** | **Sample Size** | **Mean Age (SD)** | **% Female** | **BPD Sample Characteristics** | **Measure of Dissociation** | **Consequence of Dissociation in BPD** |
| --- | --- | --- | --- | --- | --- | --- | --- | --- |
| Arntz et al., 2015  Netherlands | Randomized Controlled Trial | SCID-I  SCID-II | 86 | 30.60 (7.80) | 93% | Symptom-BPD Outpatients  Medicated | BPDSI-IV (criterion 9) | Lowers probability of recovery (interferes with effectiveness of treatment) |
| Barnow et al.,  2012  Germany | Cross-Sectional Design | SCID-II | BPD= 33  HC= 26 | BPD= 24.45(5.05)  HC= 22.65(4.87) | 100% | Symptom-BPD  Inpatients  Unmedicated | DES  DSS | Results in an elevated SCL and reduced startle response |
| Bekrater-Bodmann et al., 2015  Germany | Cross-Sectional Design | IPDE | cBPD= 29  rBPD= 19  HC= 22 | cBPD= 27.55(7.12)  rBPD= 30.89(8.13)  HC= 28.95(8.13) | 100% | Symptom-BPD  Out/Inpatients  Unmedicated | FDS  DSS-4 | Positively correlated with thermal pain threshold.  Less intense pain perception. |
| Bekrater-Bodmann et al.,  2016  Germany | Cross-Sectional Design | IPDE | cBPD= 34  rBPD= 19  HC= 25 | cBPD= 27.32(6.71)  rBPD= 29.84(5.00)  HC= 27.80(5.59) | 100% | Symptom-BPD  Out/Inpatients  Unmedicated | FDS  DSS-4 | Related to degree of body ownership/plasticity. Positively correlated with degree of illusionary limb ownership. |
| Bichescu-Burian et al.,  2017  Germany | Cross-Sectional Design | SCID-I  SCID-II  MINI | BPD-DD= 15  BPD= 13  HC= 15 | BPD-DD=  25.53(9.40)  BPD= 24.38(8.80)  HC=  23.87(6.3) | 100% | Symptom-BPD+DD  Inpatients  Medicated | DES  SCID-D  PDEQ | Decreased heart rate in response to traumatic memories. No difference in SCL. |
| Bohus et al., 2000 Germany | Cross-Sectional Design | SCID-II  DIB-R | BPD= 12  HC= 19 | BPD= 29.1(8.4)  HC= 27.3(7.8) | 100% | Symptom-BPD  Inpatients  Unmedicated | 4-Questions derived from the SDQ-5 and DES | Lowered pain perception during stress. |
| Brodsky et al., 1995  USA | Cross-Sectional Design | SCID-II | 60 | 30.00 (7.20) | 100% | Symptom-BPD  Inpatients  N/A | DES | Higher risk of self-mutilation and higher frequency of self-mutilation. |
| Cardenas-Morales et al., 2011  Germany | Cross-Sectional Design | SCID-I  SCID-II | BPD= 10  HC= 8 | BPD= 31.20(8.10)  HC= 30.00(4.40) | 100% | Symptom-BPD  Outpatients  Medicated | DSS | No correlation with pain threshold. |
| Chung et al., 2020  Germany | Cross-Sectional Design | IPDE | cBPD= 25  rBPD= 20  HC= 24 | cBPD= 27.44(6.87)  rBPD= 30.10(4.83)  HC= 27.67(5.75) | 100% | Symptom-BPD  Out/Inpatients  Medicated | DSS-4 | Positively correlated with thermal pain threshold. |
| Colle et al., 2020  Italy | Cross-Sectional Design | SCID-II | BPD= 20  HC= 20 | BPD= 29.00(9.48)  HC= 25.00(4.53) | 90% | Symptom-BPD  N/A  Medicated | DES | Positively correlated with self-harm. |
| Defrin et al., 2019  Israel | Cross-Sectional Design | Psychiatrist | BPD= 22  HC=33 | BPD= 36.40(10.00)  HC= 32.2(8.00) | 100% | Symptom-BPD  Outpatients  Medicated | DES | Positively correlated with pain threshold, it did not reach significance. |
| Demirkol et al., 2020  Turkey | Cross-Sectional Design | Psychiatrist | BPD= 74  HC=70 | BPD= 26.50(4.10)  HC= 26.8(7.90) | 67% | Symptom-BPD  Outpatients  N/A | DES | Worsens progression of BPD. |
| Didonna et al., 2019  Italy | Cross-Sectional Design | SCID-II | BPD= 48  OCD= 55  MDD= 50  HC= 49 | BPD= 36.1(12.4)  OCD= 36.10(12.4)  MDD= 50.50(11.90)  HC= 33.4(8.5) | 68% | Symptom-BPD  Outpatients  N/A | DES | Negatively correlated with mindfulness skills. |
| Ebner-Priemer et al., 2005  Germany | Cross-Sectional Design | SCID-II | BPD=21  HC= 21 | BPD= 28.50(8.10)  HC= 29.70(8.90) | 100% | Symptom-BPD  Out/Inpatients  Unmedicated | DES  DSS | Decreased startle response as assessed by EMG (Physiological blunting). |
| Ebner-Priemer et al., 2009  Germany | Cross-Sectional Design | SCID-I  IPDE | BPD=33  HC= 35 | BPD= 27.80(6.80)  HC= 28.70(7.60) | 100% | Symptom-BPD  Out/Inpatients  Unmedicated | DES  DSS | Negatively correlated with emotional learning (amygdala mediated effect). |
| Fernando et al., 2012  Germany | Cross-Sectional Design | SCID-I  SCID-II | BPD= 24  MDD= 33  HC= 41 | BPD= 26.92(5.98) MDD= 33.42(8.96) HC= 33.00(10.44) | 29% | Symptom-BPD  Inpatients  Medicated | DSS | Positively correlated with basal cortisol secretion. |
| Frankenburg et al., 2011  USA | Longitudinal Design | SCID-I  DIB-R  DIPD-R | Baseline= 213  Year 10= 200 | 27.10(5.90) | 100% | Symptom-BPD  Inpatients  Medicated | DES | Positively correlated with cBMI. |
| Haaland et al., 2009  Norway | Cross-Sectional Design | SCID-I  SCID-II | BPD-D= 10  BPD-ND= 20  HC= 30 | BPD-D= 23.70(4.80)  BPD-ND=  25.50(5.60)  HC= 25.90(6.40) | 87% | Symptom-BPD  Out/Inpatients  Medicated | DES | BPD-D patients showed reduced performance in every cognitive domain, while BPD-ND patients showed impairment in only executive functioning. |
| Hazlett et al., 2012  USA | Cross-Sectional Design | DSM-IV | BPD= 33  SPD= 28  HC= 32 | BPD= 31.60(9.10)  SPD= 35.90(11.00)  HC= 32.80(9.70) | 56% | Symptom-BPD  Outpatients  Unmedicated | DES | Negatively correlated with amygdala activation after viewing repeated unpleasant images (physiological blunting). |
| Hoerst et al., 2010  Germany | Cross-Sectional Design | SCID  IPDE | BPD= 30  HC= 30 | BPD= 29.33(7.60)  HC= 28.60(8.00) | 100% | Symptom-BPD  N/A  Unmedicated | FDS | Positively correlated with glutamate concentrations (ACC). This effect is associated with increased BPD symptom severity. |
| Hoeschel et al., 2008  Germany | Cross-Sectional Design | SCID-I  IPDE | BPD= 15  HC= 15 | BPD= 29.20(8.40)  HC= 27.30(8.60) | 100% | Symptom-BPD  Inpatients  N/A | DES | Negatively correlated with the amount of water drank (oligodipsia). |
| Irle et al., 2005  Germany | Cross-Sectional Design | SCID-I  SCID-II | BPD= 30  HC= 25 | BPD= 31.00(6.00)  HC= 33.00(7.00) | 100% | Symptom-BPD+DD  Inpatients  Medicated | DES  SCID-D | No correlation with right parietal cortex volume or parietal cortex symmetry. |
| Irle et al., 2007  Germany | Cross-Sectional Design | SCID-I  SCID-II | BPD= 30  HC= 25 | BPD= 31.00(6.00)  HC= 33.00(7.00) | 100% | Symptom-BPD+DD  Inpatients  Medicated | SCID-D | Positively correlated to right-sided precuneus size.  Dissociative disorders are positively correlated with left postcentral gyrus volume. |
| Jaeger et al., 2017  Germany | Cross-Sectional Design | ICD-10 | 103 | 28.60(9.99) | 83% | Symptom-BPD  Inpatients  N/A | FDS  DES-T | Positively correlated with symptom severity in BPD patients. |
| Johnston et al., 2009  UK | Cross-Sectional Design | SCID-II | 30 | 40.00(12.40) | 90% | Symptom-BPD  Out/Inpatients  N/A | WDS | Associated with more divisions in the BPD patients personality (dissociated dysfunctional schema model). |
| Jones et al., 1999  Wales | Cross-Sectional Design | SCID-II | BPD= 23  HC= 23 | BPD= 31.10(7.70)  HC= 31.20(8.60) | 78% | Symptom-BPD  Outpatients  Medicated | DES | Negatively correlated with specificity of recalled memories. |
| Kemperman et al., 1997  USA | Cross-Sectional Design | DSM-R | BPD-P= 26  BPD-NP=  16 | BPD-P= 29.80(6.80)  BPD-NP= 30.30(10.00) | 100% | Symptom-BPD  Inpatients  Medicated | DES | Peaks during self-harm and decreases after the end of self-harm session. |
| Kleindienst et al., 2008  Germany | Cross-Sectional Design | SCID-II  IPDE | 95 | 30.40(8.10) | 100% | Symptom-BPD  Inpatients  N/A | N/R | Self-harm to reduce dissociative symptoms. |
| Kleindienst et al., 2011  Germany | Longitudinal Design | SCID-II  DIB-R | 57 | 27.60(7.20) | 100% | Symptom-BPD  Inpatients  Medicated | DES | Showed less improvement during the DBT based treatment program. |
| Kluetsch et al., 2012  Germany | Case-Control Study | SCID-I  IPDE | BPD= 25  HC= 22 | BPD= 28.48(7.12)  HC= 28.23(8.37) | 100% | Symptom-BPD  N/A  Unmedicated | FDS  DSS | Negatively correlated with signals in posterior DMN in response to pain. |
| Kraus et al., 2009  Germany | Cross-Sectional Design | SCID-I  IPDE | BPD= 17  BPD+PTSD= 12 | BPD= 27.94(1.87)  BPD+PTSD= 27.33(1.62) | 100% | Symptom-BPD+PTSD  Out/Inpatients  Unmedicated | FDS  DSS | No significant correlation with amygdala deactivation in response to pain stimulation. |
| Krause-Utz et al., 2012  Germany | Cross-Sectional Design | SCID-CV  IPDE | BPD= 22  HC=22 | BPD= 28.18(7.02)  HC= 27.41(8.49) | 100% | Symptom-BPD  N/A  Unmedicated | DES  DSS | Negatively correlated with amygdala, insula, hippocampus, and ACC activation (physiological blunting). |
| Krause-Utz et al., 2014a  Germany | Cross-Sectional Design | SCID-I  IPDE | BPD= 22  HC=22 | BPD= 28.18(7.02)  HC= 27.41(8.49) | 100% | Symptom-BPD  Out/Inpatients  Unmedicated | DES  DSS-4 | Positively correlated with coupling of the amygdala with left insula, frontal regions, and right thalamus during emotional distractions. Positively correlated with amygdala connectivity with dlPFC during resting state. |
| Krause-Utz et al., 2014b  Germany | Cross-Sectional Design | SCID-I  IPDE | BPD= 20  HC= 17 | BPD= 29.55(7.74)  HC= 27.53(8.58) | 100% | Symptom-BPD  N/A  Unmedicated | DES | Negatively correlated with amygdala resting state functional connectivity with the cuneus, occipital lobe (V1), and the fusiform gyrus (altered gating of sensory inputs).  Positively correlated with left amygdala resting state functional connectivity with the right dlPFC (working memory, attention, inhibition control of emotions). |
| Krause-Utz et al., 2015  Germany | Cross-Sectional Design | SCID-I  IPDE | BPD= 27  HC= 26 | BPD= 27.6(7.5)  HC= 27.8(7.5) | 100% | Symptom-BPD  Out/Inpatients  Unmedicated | FDS | No significant correlation with amygdala habituation during the differential delay conditioning procedure. |
| Krause-Utz et al., 2018b  Germany | Cross-Sectional Design | SCID-I  IPDE | BPD-D= 17  BPD-ND= 12  HC= 18 | BPD-D= 27.41(6.20)  BPD-ND= 25.17(6.21)  HC= 29.61(8.61) | 100% | Symptom-BPD  Out/Inpatients  Medicated | DES  DSS-4 | Associated with working memory impairments, bilateral amygdala deactivation, and lower activity in the left cuneus, lingual gyrus, and the posterior cingulate cortex.  Negatively correlated with amygdala functional connectivity with fusiform gyrus, and positively correlated with amygdala functional connectivity with the right middle/superior temporal gyrus and left parietal lobe (emotional salience and working memory/dampening effects). |
| Krause-Utz et al., 2019  Germany | Cross-Sectional Design | SCID-I  IPDE | BPD= 37  BPD+PTSD= 20  HC= 27 | BPD= 31.28(10.28)  BPD+PTSD= 34.16(9.92)  HC= 28.88(6.39) | 100% | Symptom-BPD+PTSD  Out/Inpatients  Medicated | DES  DSS-4 | Positively correlated with an increase in high-frequency heart rate variability (slowed heart rate) |
| Kremers et al., 2004  Netherlands | Cross-Sectional Design | SCID-II | BPD-MDD= 47  BPD= 36  HC= 30 | BPD-MDD= 29.80(8.10)  BPD= 31.80(7.80)  HC= 34.70(7.40) | 88% | Symptom-BPD  Outpatients  N/A | DES | No correlation with specificity of autobiographical memory. |
| Lange et al., 2005  Germany | Cross-Sectional Design | SCID-I  SCID-II | BPD= 17  HC= 9 | BPD= 32.00(4.00)  HC= 33.00(6.00) | 100% | Symptom-BPD  Inpatients  Medicated | DES  SCID-D | Negatively correlated with resting metabolic rate of the left-sided precuneus and posterior cingulate cortex.  Positively correlated with activation of the right precuneus (interpersonal difficulties). |
| Loffler et al., 2020  Germany | Cross-Sectional Design | IPDE | cBPD= 26  rBPD= 22  HC= 20 | cBPD= 31.65(9.09)  rBPD= 29.77(5.44)  HC= 27.05(7.17) | 100% | Symptom-BPD  Out/Inpatients  Medicated | FDS | Negatively correlated with feelings of body ownership. |
| Ludascher et al., 2007  Germany | Cross-Sectional Design | SCID-II  DIB-R | BPD= 12  HC= 12 | BPD= 20.00(9.00)  HC= 29.00(6.00) | 100% | Symptom-BPD  N/A  Unmedicated | FDS  DSS | Positively correlated with pain threshold. |
| Ludascher et al., 2009  Germany | Cross-Sectional Design | IPDE | BPD-SH= 13  BPD-NSH= 11  HC= 24 | BPD-SH= 28.00(8.00)  BPD-NSH= 30.00(7.00)  HC= 24.00(4.00) | 100% | Symptom-BPD  Out/Inpatients  Unmedicated | DES  DSS | Positively correlated with self-harm. |
| Ludascher et al., 2010  Germany | Cross-Sectional Design | IPDE | BPD= 15  BPD+PTSD= 10 | BPD= 28.10(7.60)  BPD+PTSD= 28.80(8.20) | 100% | Symptom-BPD+PTSD  N/A  Unmedicated | FDS  DSS | Negatively correlated with pain sensitivity.  Associated with fronto-limbic activation. |
| McGirr et al., 2009  Canada | Retrospective Observational Study | SCID-I  SCID-II | BPD-S= 62  BPD= 35 | BPD-S= 36.14(10.94)  BPD= 38.34(11.11) | 17% | Symptom-BPD  N/A  N/A | SCID-II | Negatively correlated with suicide (protective). |
| Merckelbach et al., 2005  Netherlands | Cross-Sectional Design | Psychiatrist | BPD= 20  SCZ= 22  MDD= 19 | BPD= 38.20(8.40)  SCZ= 38.90(11.50)  MDD= 47.20(10.00) | 38% | Symptom-BPD  Outpatients  Medicated | DES  DES-T | Positively correlated with fantasy proneness. |
| Miller et al., 1993  USA | Retrospective Observational Study | Psychiatrist | BPD-SA= 52  BPD= 40 | BPD-SA= 29.00(7.00)  BPD= 29.00(6.00) | 81% | Symptom-BPD  Inpatients  Medicated | DSM-3 | Positively correlated with no substance abuse and alcohol-sedative drug abuse. |
| Navarro-Haro et al., 2015  Spain | Cross-Sectional Design | SCID-II | 68 | N/A | 100% | Symptom-BPD  Outpatients  Medicated | DES-II | Positively correlated with increased self-harm in BPD-ED patients. |
| Niedtfeld et al., 2013 Germany | Cross-Sectional Design | SCID-I  IPDE | BPD= 32  BPD+PTSD= 21  HC= 60 | BPD= 29.67(8.06)  HC= 28.5(7.49) | 100% | Symptom-BPD+PTSD  N/A  Unmedicated | FDS | Positively correlated with gray matter volume in middle temporal gyrus |
| Paret et al., 2016  Germany | Cross-Sectional Design | SCID  IPDE | BPD= 21  HC= 15 | BPD= 27.00(6.70)  HC= 25.10(3.70) | 100% | Symptom-BPD  Outpatients  Unmedicated | DSS-4 (Short Version) | Negatively correlated with performance on the acquisition component of the operant conditioning task. |
| Pec et al., 2021  Czech Republic | Longitudinal Design | SCID-II  ICD-10 | BPD= 105 | BPD= 31.20(8.40) | 77% | Symptom-BPD  Outpatients  Medicated | DES | Positively correlated with treatment drop out. No significant correlation with treatment effectiveness. |
| Popkirov et al., 2019  Germany | Cross-Sectional Design | DSM-5 | BPD= 26  HC= 26 | BPD= 31.42(9.75)  HC= 27.65(6.27) | 92% | Symptom-BPD  Inpatients  Medicated | DES | Positively correlated with frontal EEG asymmetry before mood induction task (leftward asymmetry). |
| Prossin et al., 2010  USA | Cross-Sectional Design | SCID-I  SCID-II | BPD= 18  HC= 14 | BPD= 28.00(9.00)  HC= 35.00(10.00) | 100% | Symptom-BPD  N/A  Unmedicated | DES | Negatively correlated with the nondisplaced binding potential in the right caudate region. Correlation did not persist after correction for multiple comparisons. |
| Renneberg et al., 2005  Germany | Cross-Sectional Design | SCID-I  SCID-II | BPD= 30  MDD= 27  HC= 30 | BPD= 28.5(9.10)  MDD= 39.10(8.00)  HC= 28.40(8.60) | 100% | Symptom-BPD  Inpatients  N/A | DES | Not correlated with specificity of recall of autobiographical memory. |
| Rusch et al., 2007  Germany | Cross-Sectional Design | DSM-IV | BPD= 20  HC= 20 | BPD= 28.0(5.9)  HC=27.2(7.6) | 100% | Symptom-BPD  Outpatient  Unmedicated | FDS | Positively correlated with mean diffusivity in inferior frontal white matter. |
| Russ et al., 1993  USA | Cross-Sectional Design | SCID-II | BPD-NP= 13  BPD-P= 14 | BPD-NP= 30.00(8.80)  BPD-P= 29.00(6.20) | 100% | Symptom-BPD  Inpatients  N/A | DES | Negatively correlated with pain perception during self-harm. |
| Russ et al., 1996  USA | Cross-Sectional Design | SCID-II | BPD-NP= 15  BPD-P= 24  HC= 22 | BPD-NP= 28.40(7.90)  BPD-P= 31.60(8.00)  HC= 33.90(9.90) | 100% | Symptom-BPD  Inpatients  Medicated | DES | Negatively correlated with the ability to discriminate between imaginary painful and mildly painful situations (pain insensitive). |
| Russ et al., 1999  USA | Cross-Sectional Design | SCID-II | BPD-NP= 19  BPD-P= 22  MDD= 15  HC= 20 | BPD-NP= 25.80(5.70)  BPD-P= 31.10(8.90)  MDD= 33.30(9.80)  HC= 30.10(6.60) | 100% | Symptom-BPD  Inpatients  Medicated | DES | Negatively correlated with pain perception (analgesic effect).  Positively correlated with theta activity. |
| Schmahl et al., 2013  Germany | Longitudinal Design | SCID-I | 31 | 27.47(7.95) | 100% | Symptom-BPD  Inpatients  Medicated | DSS | Positively correlated with IL-6 gene expression.  Negatively correlated with GNAS, MAPK1, MAPK3, MAPK8, GNAI2, ARRB1, ARRB2, RGS2, IL1B, NR3C1, CREB1, PREP, SLC18A24. |
| Semiz et al., 2008  Turkey | Cross-Sectional Design | SCID-I | BPD= 88  HC= 100 | BPD= 21.70(3.60)  HC= 22.30(3.90) | 44% | Symptom-BPD  Inpatients  Unmedicated | DES | Positively correlated with dream anxiety. |
| Shearer, 1994  USA | Cross-Sectional Design | SCID-II | 62 | 25.02(21.19) | 100% | Symptom-BPD+DD  Inpatients  N/A | DES | Positively correlated with behavioural dyscontrol, self-harm, alcohol abuse, and PTSD symptoms. |
| Simeon et al., 2003  USA | Cross-Sectional Design | SCID-I  SIDP | BPD= 20  HC= 24 | BPD= 37.90(12.60)  HC= 35.50(11.70) | 43% | Symptom-BPD  Outpatients  Unmedicated | DES | Positively correlated with primitive defensive structures and fearful attachments. |
| Simeon et al., 2007  USA | Cross-Sectional Design | SCID-P  SID-P | BPD= 13  HC=11 | BPD= 39.20(10.60)  HC= 27.10(6.20) | 46% | Symptom-BPD  Outpatients  Unmedicated | DES | Markers of higher biological vulnerability to stress (norepinephrine and cortisol). |
| Spinhoven et al., 2006  Netherlands | Longitudinal Design | SCID-II | BPD-MDD= 37  BPD= 18 | BPD-MDD= 29.80(8.60)  BPD= 31.70(7.90) | 91% | Symptom-BPD  Outpatients  Medicated | DES | No correlation with autobiographical memory. |
| Stevens et al, 2004  Germany | Cross-Sectional Design | IBPD | BPD= 22  HC= 25 | BPD= 31.90(9.10)  HC= 30.50(10.40) | 100% | Symptom-BPD  Out/Inpatients  Medicated | FDS | No correlation with working memory. |
| Wedig et al., 2012  USA | Longitudinal Design | SCID-I  DIB-R  DIPD-R | Baseline= 264  End= 231 | 26.90(5.80) | 88% | Symptom-BPD  Inpatients  N/A | DES | Positively correlated with risk of suicide attempt. |
| Wingenfeld et al., 2009  Germany | Cross-Sectional Design | DSM-IV | BPD= 20  HC= 20 | BPD= 29.75(13.2)  HC= 29.45(12.4) | 70% | Symptom-BPD  Inpatients  Medicated | DSS | No significant correlation with performance on EST and BOLD signals. |
| Winter et al., 2015  Germany | Cross-Sectional Design | SCID-I  IPDE | BPD-D= 18  BPD= 19  HC= 19 | BPD-D= 27.61(5.95)  BPD= 28.05(7.82)  HC= 28.74(8.07) | 100% | Symptom-BPD  N/A  Unmedicated | DES  DSS-4 | Induced dissociation was correlated with inefficient cognitive inhibition during EST. |
| Wolf et al., 2011  Germany | Cross-Sectional Design | SCID | BPD= 17  HC= 17 | BPD= 28.60(7.30)  HC= 27.20(8.0) | 100% | Symptom-BPD  Out/Inpatients  Medicated | DSS | Positively correlated with abnormal connectivity in the insula of the default mode network (pain sensitivity). |
| Wolf et al., 2012  Germany | Cross-Sectional Design | SCID-I  SCID-II | BPD= 16  HC= 16 | BPD= 27.80(6.40)  HC= 27.60(8.10) | 100% | Symptom-BPD  Out/Inpatients  Medicated | DSS | No correlation with orbitofrontal cortex perfusion. |
| Zanarini et al., 2011  USA | Longitudinal Design | SCID-I  DIB-R  DIPD-R | Baseline= 271  End= 249 | 26.90(5.80) | 86% | Symptom-BPD  Inpatients  Medicated | DES | Positively correlated with self-harm overtime. |

*Abbreviations:* ACC, anterior cingulate cortex; ARRB2, Beta-arrestin-2; BPD, Borderline Personality Disorder; BPD+DD, Borderline Personality Disorder with a comorbid Dissociative Disorder; BPD+PTSD, Borderline Personality Disorder with comorbid Posttraumatic Stress Disorder; BPD-D, Borderline Personality Disorder with Induced Dissociation; BPD-DD, Borderline Personality Disorder with a comorbid Dissociative Disorder; BPD-MDD, Borderline Personality Disorder with comorbid Major Depressive Disorder; BPD-ND, Borderline Personality Disorder without Induced Dissociation; BPD-NP, Borderline Personality Disorder who do not experience pain during self-harm; BPD-NSH, Borderline Personality Disorder without Self-harm; BPD-P, Borderline Personality Disorder who experience pain during self-harm; BPD-S, Borderline Personality Disorder patients who died by suicide; BPD-SH, Borderline Personality Disorder with Self-harm; BPDSI-IV, Borderline Personality Disorder Severity Index- version 4; cBMI, Cumulative Body Mass Index; cBPD, Current Borderline Personality Disorder; CREB1, Cyclic AMP-responsive element-binding protein; DES, Dissociative Experience Scale; DES-T, Dissociative Experience Scale- Taxon; DIB-R, Revised Diagnostic Interview for Borderlines; DIPD-R, Diagnostic Interview for DSM-III-R Personality Disorders; DSM-3, Diagnostic and Statistical Manual of Mental Disorders- Third Edition; DSM-5, Diagnostic and Statistical Manual of Mental Disorders- Fifth Edition; DSM-IV, Diagnostic and Statistical Manual of Mental Disorders- Fourth Edition; DSM-R, Diagnostic and Statistical Manual of Mental Disorders-Revised; DSS, Dissociative Tension Scale; EEG, Electroencephalogram; EMG, Electromyography; EST, Emotional Stroop Test; FDS, Fragebogen zu Dissoziativen Symptomen; GNAI2, Guanine nucleotide-binding protein G(i) subunit alpha-2; ARRB1, Beta-arrestin-1; GNAS, Guanine nucleotide-binding protein G(s) subunit alpha isoforms; HC, Healthy Control; IBPD, Diagnostic Interview for Borderline Personality Disorder; ICD-10, Classification of Disease; IL1B, Interleukin-1B; IL-6, Interleukin-6; IPDE, International Personality Disorder Examination; MAPK1, Mitogen-activated protein kinases 1; MAPK3, Mitogen-activated protein kinases 3; MAPK8, Mitogen-activated protein kinases 8; MDD, Major Depressive Disorder; MINI, The Mini International Neuropsychiatric Interview; N/A, Not Applicable (was not reported by authors); ND, No Induced Dissociation; NR3C1, Glucocorticoid receptor; OCD, Obsessive Compulsive Disorder; PDEQ, Peritraumatic Dissociative Experience Scale; PREP, Prolylen-dopeptidase; rBPD, Remitted Borderline Personality Disorder; RGS2, Regulator of G-protein signaling 2; SCID-CV, Structured Clinical Interview for the DSM- Axis 1 Disorders- Clinical Version; SCID-D, Structured Clinical Interview for the DSM- Dissociative Disorders; SCID-I, Structured Clinical Interview for the DSM- Axis 1 disorders; SCID-II, Structured Clinical Interview for the DSM- Axis two disorders; SCID-P, Structured Clinical Interview for the DSM- Personality Disorders; SCL, Skin Conductance Level; SCZ, Schizophrenia; SIDP, and the Structured Interview for DSM Personality Disorders; SID-P, Structured Interview for DSM Personality Disorders; SLC18A24, Synaptic vesicular amine transporter; SPD, Schizotypal Personality Disorder; SPQ-5, Somatoform Dissociation Questionnaire; WDS, Wessex Dissociation Scale.

**Table 1b:** Study interventions, scores, and main findings.

| **Study** | **Intervention** | | **Outcome** | |
| --- | --- | --- | --- | --- |
|  | Objective | Intervention | Measures and Mean (SD) Scores | Main Results |
| Arntz et al., 2015  Netherlands | Experiment 1: To assess factors that may affect treatment response.  Experiment 2: To assess the presence and impact of dissociation present during therapy session. | Patients were treated using Schema Therapy (ST) and Transference Focused Psychotherapy (TFP) for 3-years. BPD symptomology was assessed every three months using the BPDSI-IV. Audiotapes of treatment sessions were analyzed to assess the effect dissociation had on treatment effectiveness. | BPDSI-IV =33.97(7.97) | Experiment 1: High dissociation was associated with poorer treatment response. Seventy-three percent and 47% of participants with low dissociation responded to ST and TFP respectively. Response rate dropped to 32% (ST) and 14% (TFP) in patients with high levels of dissociation.  Experiment 2: In-session dissociation was negatively correlated with treatment effectiveness. |
| Barnow et al.,  2012  Germany | To assess the moderating role of dissociation during emotion regulation. | Three idiographic aversive scripts and 3 neutral scripts were presented to the participants followed by a period where participants are asked to imagine the script they just read. A startle probe was used during the image phase and the intertrial interval. State dissociation, measured using the DSS was assessed before the intervention and then again after completion. | DES:  BPD= 21.04 (13.35)  HC= 8.29 (7.81)  DSS:  BPD-Pretest= 0.77 (0.67)  BPD-Posttest= 1.32 (1.04)  HC-Pre= 0.09 (0.13)  HC-Post= 0.24 (0.19) | Increased scores on the DSS was correlated with elevated skin conductance levels and a reduced startle response, suggesting that dissociative states during emotional tasks reduce emotional and physiological reaction. |
| Bekrater-Bodmann et al., 2015  Germany | To assess if reduced pain sensitivity observed in BPD is due to an altered evaluation of pain and/or altered sensory integration. | A thermal grill illusion (TGI) that alternates between warm and cold stimuli (16^o^C-40^o^C) was used.  To assess pain threshold, the Thermal Sensory Analyzer was used (0^o^C-52^o^C). | DSS-4= NR  FDS= NR | State dissociation in current BPD patients, as measured by the DSS-4, was negatively correlated with the magnitude of the TGI (r=-0.54, p=0.032). A similar, nonsignificant trend was observed with trait dissociation (measured using the FDS; r=-0.36, p=0.19).  Current BPD patients have a lower pain threshold compared to remitted BPD patients and healthy controls. Thermal perception did not differ. |
| Bekrater-Bodmann et al.,  2016  Germany | To assess the degree of body plasticity experienced in BPD patients. | A rubber hand illusion (RHI) was used. This task had participants place both hands flat on a table with their right hand in view and their left hand hidden behind a screen. A life-like rubber left hand was also placed in their line of view. Both left hands were stimulated simultaneously using a cotton swab while participants were directed to attend to the rubber left hand. The left hands were either stroked in the same direction or opposite directions. | DSS-4= NR  FDS= NR | The RHI was positively correlated with measures of state (r=0.30, p=0.049) and trait (r=0.32, p=0.043) dissociation.  BPD patients who experience dissociation experience more body plasticity. |
| Bichescu-Burian et al.,  2017  Germany | To evaluate the impact peritraumatic dissociation (PD) has on dissociative patterns of reaction on BPD patients. | Participants were retrospectively interviewed, and three individualized scripts were created.  A psychophysiological experiment took place where the three scripts were presented. After each script the participant was asked to imagine the experience detailed in the script. Participants' heart rate and skin conductance level was recorded throughout the experiment. | DES:  BPD+PD= 34.66 (20.9)  BPD= 15.91 (11.8)  HC= 4.90 (2.5)  PDEQ:  BPD+PD= 2.71(0.7)  BPD=1.21(0.2)  HC=0.86(0.6) | BPD participants with high levels of PD showed a decrease in heart rate. Whereas BPD participants with low PD and healthy controls showed increased heart rate. No difference in skin conductance levels were observed. |
| Bohus et al., 2000 Germany | To determine if participants with BPD a) experienced less pain than healthy controls during periods of self-reported calmness, b) experienced less pain during periods of self-reported distress. | Health controls underwent the Cold Pressor Test (CPT) and the Tourniquet Pain Test (TPT) while calm. Participants with BPD underwent the CPT and TPT tests while calm and again while distressed.  Dissociation was assessed using four questions before and after the tests. | Numbness:  BPD-Calm= 0.75 (2.95)  BPD-Distress= 2.67(2.84)  HC= 0.21(0.54)  Anesthesia:  BPD-Calm= 0.42(1.00)  BPD-Distress= 3.58(2.54)  HC= 0.16(0.50) | Participants with BPD experienced significantly less pain during periods of self-reported calmness than healthy controls. During periods of self-reported distress, participants with BPD experienced significantly less pain than during periods of calmness.  Participants with BPD experienced more dissociative symptoms (numbness and anesthesia) during periods of distress than calmness. |
| Brodsky et al., 1995  USA | To identify the prevalence of dissociation and its relationship with self-mutilation and childhood abuse in female inpatients with BPD. | Participants were assessed using a treatment history interview, the DES, the SEQ, and the HDRS. | DES= 19.58 (16.36) | Higher DES scores were associated with a higher risk of self-mutilation and higher frequency of self-mutilation (r=0.41, p=0.001). |
| Cardenas-Morales et al., 2011  Germany | To assess the affective components of pain in BPD. | Used repetitive peripheral magnetic stimulation (rPMS) to administer bursts that gradually increased in intensity by 10% until pain was detected by the participant. | DSS=NR | In BPD patients, pain threshold was positively correlated with motivation to engage in non-suicidal self-harm. No correlation between a measure of state dissociation and pain threshold was observed. |
| Chung et al., 2020  Germany | To identify if remitted BPD patients are prone to dissociation and pain hyposensitivity. | Participants listened to individualized stressful and neutral scripts through the script-driven imagery approach. Dissociation was assessed before (pre-induction) and after (post-induction) the scripts were listened to. Additionally, heat pain threshold (HPT) was assessed after listening to the scripts. | DSS-4  Pre-induction:  cBPD-  Neutral script= 1.44(1.73)  Stress script= 1.15(1.46)  rBPD-  Neutral= 0.16(0.33)  Stress= 0.19(0.52)  HC-  Neutral= 0.03(0.15)  Stress= 0.04(0.16)  Post-induction:  cBPD-  Neutral= 1.30(1.75)  Stress= 2.46(2.16)  rBPD-  Neutral=0.19(0.49)  Stress= 1.01(1.6)  HC-  Neutral= 0.05(0.15)  Stress= 0.05(0.16) | Following the neutral script, participants currently diagnosed with BPD had the highest scores on the DSS-4 and a HPT, and healthy controls had the lowest. Remitted BPD participants were in between.  Following the stressful script, remitted BPD patients showed high HPT that was comparable to participants currently diagnosed with BPD.  DSS-4 scores were positively correlated with HPT in the currently diagnosed with BPD group (r=0.40, p=0.047). No significant correlation was seen in the remitted BPD group (r=0.22, p=0.36) and healthy controls (r=-0.37, p=0.08). |
| Colle et al., 2020  Italy | To evaluate if dissociation is associated with a decreased sense of agency, and whether self-harm is a strategy used to regain agency and reduce dissociative stress. | Participants' sense of agency was evaluated using the Sensory Attenuation paradigm. This paradigm contains two experimental conditions: self-generated electrical stimulation and the other-generated electrical stimulation. Both conditions were of equal intensity. Following each trail, participants rated the perceived intensity of the stimulus on a 0-7 point Likert Scale. | DES:  BPD-NSSI= 12.96(10.57)  BPD+NSSI= 31.12(15.76)  HC= 9.98(6.27)  Stimulus Intensity (Likert Scale)  BPD-NSSI:  Self-generated= 4.95(1.2)  Other-generated= 4.41(1.69)  BPD+NSSI:  Self-generated= 4.62(1.2)  Other-generated= 4.92(1.08)  HC:  Self-generated= 5.04(0.75)  Other-generated= 5.23(0.67) | Healthy controls and BPD participants who engage in self-harm, rated the self-generated electrical stimulation as less intense than the other-generated electrical stimulation. Conversely, BPD participants who do not self-harm showed an opposite effect.  BPD participants who engage in self-harm had higher DES scores than the other two groups. |
| Defrin et al., 2019  Israel | To test if reduced pain sensitivity in BPD is specific to pain or is generalized to innocuous stimuli. | Participants thresholds for warm sensations (WST), HPT, conditioned pain modulation (CPM), and temporal summation heat-pain (TSP) were assessed. Pain was stimulated using a Peltier-based computerized thermal stimulator and a 10-L water bath. | DES:  BPD= 33.5(18)  HC= 9.2(7) | BPD patients experienced decreased sensitivity to noxious and innocuous thermal stimuli. Decreased sensitivity was not correlated with DES scores in BPD.  In the healthy controls, DES scores were positively correlated with WST; it did not reach statistical significance. |
| Demirkol et al., 2020  Turkey | To assess the effect anxiety sensitivity and depressive symptoms have on dissociation and perceived stress. | Participants completed a sociodemographic data form, the BDI, the DES, the Perceived Stress Scale, and the Anxiety Sensitivity Index. | DES:  BPD= 39 (range: 27-52)  HC= 5 (range: 3-9) | The presence of dissociative experiences were associated with worse progression of BPD. Higher DES scores were significantly correlated with perceived stress (r=0.433, p<0.001), anxiety sensitivity (r=0.652, p<0.001), and depressive symptoms (r=0.749, p<0.001). |
| Didonna et al., 2019  Italy | To evaluate mindfulness in participants with OCD, MDD, BPD, or healthy controls. | Participants completed a clinical assessment (FFMQ, SCL-90-R, DES, Alexithymia Scale 20, BDI-II) | DES:  BPD= 28.4(18.5)  MDD=16.9(15.4)  OCD=12.3(13)  HC=6.1(4.2) | Participants with an OCD, MDD, or BPD diagnosis showed deficits in mindfulness compared to controls.  Higher DES scores were negatively correlated with mindfulness skills. |
| Ebner-Priemer et al., 2005  Germany | To assess the amygdala mediated startle response in BPD patients. | Participants listened to 15 startle tones (95-dB, 500ms, 1000 Hz) while their heart rates, skin conductance, and orbicularis oculi EMG response were measured. | DES:  BPD= 22.8(7.75)  HC= 3.3(2.74)  DSS:  BPD= 1.38(1.25)  HC= 0.07(0.14) | State dissociation in BPD patients, as measured by the DSS, was negatively correlated with their EMG response (r=-44, p=0.068) indicating a decreased startle response. Trait dissociation did not show a significant correlation (measured using the DES; r=-0.10, p=0.734). |
| Ebner-Priemer et al., 2009  Germany | To ascertain whether state dissociation would affect emotional learning, but not declarative learning. | An aversive differential delay conditioning procedure was conducted. The procedure included 4 phases: the habituation phase, the acquisition phase, and the early and late extinction phases. A baby's cry was used as the aversive unconditioned stimuli and 2 neutral inkblots were the conditioned stimuli. | DES:  BPD-D= 15.9(11.5)  BPD+D= 35.5(12.7)  HC= 3.32(3.01)  DSS:  BPD-D= 0.12(0.11)  BPD+D= 3.10(1.6)  HC= 0.12(0.22) | Participants with high levels of state dissociation, measured using the DSS, had less acquisition of differential delay conditioning than participants with low state dissociation and healthy controls. Additionally, they did not experience an increase in arousal, emotional valence, or skin conductance during the conditioned stimuli, whereas the low state dissociation BPD group and healthy controls did.  State dissociation was negatively correlated with emotional learning (amygdala mediated effect). |
| Fernando et al., 2012  Germany | To examine the impact HPA-axis abnormalities have on clinical characteristics in BPD, MDD, and healthy controls. | The dexamethasone suppression test (0.5mg) was used to assess basal cortisol release and HPA-axis feedback sensitivity (salivary cortisol). | DSS:  BPD= 24.97(16.97)  MDD=17.53(11.76)  HC=3.96(4.73) | Higher DSS scores were positively correlated with basal cortisol secretion. |
| Frankenburg et al., 2011  USA | To examine the relationship between cumulative body mass index (cBMI) and psychosocial, medical, and symptomatic factors associated with BPD. | Two-hundred and thirteen participants were initially measured, weighed, and interviewed (baseline). Additionally, participants attend follow-up sessions at 6, 8, and 10 years. | DES= NR | At baseline, the DES was not administered. At year 6, 23 out of the 213 participants scored 30 or above on the DES (10.8%). At year 8, 15 out of the remaining 205 participants scored 30 or above (7.3%). A year 10, 15 out of the 200 remaining participants scored 30 or above (7.5%).  At the end of the study, DES scores of 30 or above were associated with a 43% increase in cBMI. |
| Haaland et al., 2009  Norway | To assess the impact pathological dissociation has on neuropsychological functioning. | Participants completed an array of different cognitive tests that assessed their executive functioning, attention, long-term verbal memory, working memory, and general cognition. | DES (total score):  BPD= 14.9(9)  BPD+D= 38.5(15.5)  HC= 2.2(1.9)  DES (pathological dissociation score):  BPD= 10.5(7.2)  BPD+D= 36.3(19.8)  HC= 0.5(0.9) | BPD participants who experience pathological dissociation showed reduced performance in every cognitive domain, while BPD participants who do not experience pathological dissociation showed impairment in only executive functioning. |
| Hazlett et al., 2012  USA | To assess amygdala activity in response to repeated emotional pictures in BPD, schizotypal personality disorder (SPD), and healthy controls. | Participants were shown a mix of neutral, unpleasant, and pleasant pictures. During the task, an fMRI image was taken for each participant. | DES=NR | Overall, BPD patients experienced greater amygdala potentiation in response to repeated emotional pictures.  DES scores were negatively correlated with amygdala activation after viewing repeated unpleasant images (BPD: r=-0.36, p=0.047; SPD: r=-0.40, p=0.042). |
| Hoerst et al., 2010  Germany | To identify if the anterior cingulate cortex (ACC) metabolism is altered in BPD, and its impact on BPD pathology. | Participants' brains were imaged using a proton MRS. Self-reports of participants' impulsivity and BPD symptom severity were collected. | FDS:  BPD= 23.69 (12.3)  HC= 2.91 (3.4) | In BPD, increased glutamate concentration in the ACC was positively correlated with impulsivity (r=0.508, p=0.009), cognitive impulsivity (r=0.358, p=0.08) and FDS scores (r=0.43, p=0.03). |
| Hoeschel et al., 2008  Germany | To identify the frequency of fluid intake (oligodipsia) in BPD, and the impact dissociation has on oligodipsia. | The amount of fluid consumed was measured daily for seven days through self-report measures. Additionally, 24 hour urine volume and osmolality was measured. | DES:  BPD= 33.5(19.4)  HC= 3.7(2.4)  Daily Fluid intake:  BPD= 1492(659)  HC= 2521(695) | Participants with BPD drank significantly less water than healthy controls.  DES scores were negatively correlated with the amount of oligodipsia (r=-0.762, p<0.001) and urine osmolality (r=-0.665, p<0.001). |
| Irle et al., 2005  Germany | To assess the volume of the parietal cortex and hippocampus in BPD compared to healthy controls. | Participants' brains were scanned using an MRI machine. | DES:  BPD= 24.7(12.3)  HC=2.1(4)  SCID-D:  14%= lifetime depersonalization  77%= current depersonalization  3%= Lifetime dissociative amnesia  20%= current dissociative amnesia  13%= current DID  37%= PTSD | BPD participants' right parietal cortex was 11% smaller than healthy controls, and their hippocampus was 17% smaller. BPD participants had increased leftward asymmetry compared to healthy controls.  DES scores were not correlated with right parietal cortex volume or parietal cortex asymmetry. |
| Irle et al., 2007  Germany | To evaluate the volume of the superior and inferior parietal cortices in BPD patients who experienced severe childhood sexual and physical abuse compared to healthy controls. | Participants' brains were scanned using an MRI machine. | SCID-D:  90%= lifetime/current depersonalization  23%= Lifetime/current dissociative amnesia  13%= current DID  37%=PTSD | BPD patients had a 9% smaller right-sided precuneus compared to healthy controls. BPD patients with a comorbid dissociative disorder had a larger postcentral gyrus compared to healthy controls (+13%) and BPD participants without a dissociative disorder (+11%) (p<0.001).  Depersonalization was positively correlated to right-sided precuneus size (r=0.469, p=0.010). |
| Jaeger et al., 2017  Germany | To assess the co-occurrence of dissociation with BPD symptoms. | Participants completed the following clinical measures: BSL-95, FDS, and SCL-90-R. | FDS= 24.66 (18.56)  DES-T= 28.31 (19.32) | Measures of trait dissociation were positively correlated with symptom severity in BPD patients. |
| Johnston et al., 2009  UK | To evaluate the association between dissociation, childhood trauma, and dysfunctional schema model in BPD participants. | Participants completed the following clinical measures: WDS, CTQ, GHQ, and SMQ. | WDS= 2.76(0.75) | The dysfunctional schema modes “Angry and Impulsive Child” and “Abandoned and Abused Child” predict dissociation severity in BPD patients.  Higher WDS scores were associated with more divisions in the BPD patient’s personality. |
| Jones et al., 1999  Wales | To assess whether BPD participants overgeneralize their autobiographical recall, and if this overgeneralization is associated with dissociation. | Participants completed the autobiographical memory test (AMT). This test prompted participants to recall a specific memory within 30-seconds that is related to a presented cue word. | DES:  BPD= 39.9(17)  HC= 8.9(7.3) | Des scores were negatively correlated with specificity of recalled memories (more vague memories). |
| Kemperman et al., 1997  USA | To assess the mood regulatory capacity of self-harm in BPD. | Participants were divided into two groups, those who felt pain during self-harm (BPD-P) and those who did not (BPD-NP). Participants completed visual analog scales. | DES:  BPD+P= 25.8(16.2)  BPD-NP= 43.6(19.2) | Participants reported feeling better after self-harm. Dissociation peaks during self-harm and decreases after self-harm (F=9.7, df=2.36, p=0.000). BPD-NP participants had higher DES scores than the BPD-P group. |
| Kleindienst et al., 2008  Germany | To identify motives behind self-harm. | Participants completed a self-rating questionnaire on self-harm (QNSSI). | N/A | Self-harm was primarily used to reduce aversive tension (51% of patients). Self-harm was also used to reduce unpleasant emotions (13%) and as a form of self-punishment (12%). Additionally, some participants engaged in self-harm to reduce dissociative symptoms (8%=recover control; 7%=regain awareness of physical sensations; 7%=regain sense of reality). |
| Kleindienst et al., 2011  Germany | To identify whether dissociation impacts treatment response to Dialectical Behavioural Therapy (DBT) in BPD inpatients. | BPD inpatients underwent three months of DBT. | DES= 25.8(16.7)  DES range= 2.1-61.8 | Higher DES scores were associated with less improvement during DBT treatment sessions (β=-0.017±0.006, p=0.008). |
| Kluetsch et al., 2012  Germany | To assess the relationship between default mode network (DMN) connectivity and altered pain processing in BPD patients. | Participants underwent psychophysical assessments and fMRI while experiencing painful heat stimulation and neutral temperature stimulation. | FDS:  BPD= 20.81(10.17)  HC= 3.38(2.48)  DSS:  BPD= 0.65(0.82)  HC= 0.13(0.32) | Pain related connectivity in the posterior DMN was positively correlated with trait dissociation (measured using the FDS; r=0.59) and BPD symptom severity (r=0.63) in response to pain. |
| Krause et al., 2009  Germany | To assess if amygdala deactivation is a neural correlate of pain processing in BPD with comorbid PTSD (BPD+PTSD). | Participants underwent an fMRI scan while experiencing heat stimulation with an individually adapted temperature they perceived deserved a 40% pain rating (40^o^C-48^o^C). | FDS:  BPD= 20.48(2.62)  BPD+PTSD= 20.99(3.23)  DSS:  BPD= 1.16(0.28)  BPD+PTSD= 1.22(0.31) | There was no apparent difference in pain sensitivity between the BPD only group and the BPD+PTSD group. Amygdala deactivation was more prominent in the BPD+PTSD group than the BPD only group. Measures of state and trait dissociation and symptom severity were not correlated with amygdala deactivation. |
| Krause-Utz et al., 2012  Germany | To evaluate the impact emotional distraction has on working memory in BPD participants. | Participants performed a Sternberg working memory task while observing negative emotional pictures and neutral pictures. Participants' brains were imaged using an fMRI during the task. | DES:  BPD= 30.85(15.27)  HC= 2.45(1.89)  DSS-4  Pre-fMRI:  BPD= 1.97(1.73)  HC= 0.10(0.20)  Post-fMRI:  BPD= 2.97(2.25)  HC= 0.13(0.26) | BPD participants had longer reaction times and increased activation in the amygdala and insula when viewing distracting emotional pictures compared to healthy controls.  DES and DSS-4 scores were negatively correlated with amygdala, insula, hippocampus, and ACC activation (physiological blunting). |
| Krause-Utz et al., 2014a  Germany | To further evaluate the impact emotional distraction has on working memory in BPD participants. | Data gathered from the Kraus-Utz et al, 2012 experiment was further analyzed. | DES:  BPD= 30.85(15.27)  HC= 2.45(1.89)  DSS-4  Pre-fMRI:  BPD= 1.97(1.73)  HC= 0.10(0.20)  Post-fMRI:  BPD= 2.97(2.25)  HC= 0.13(0.26) | DES and DSS-4 scores were positively correlated with coupling of the amygdala with left insula, frontal regions, and right thalamus during emotional distractions. Positively correlated with amygdala connectivity with dlPFC during resting state. |
| Krause-Utz et al., 2014b  Germany | To assess resting-state functional connectivity of the core fronto-limbic regions associated with BPD. | Participants' brains were imaged using an fMRI machine. | DES:  BPD= 32.25(15.78)  HC= 2.21(1.69) | DES scores were negatively correlated with amygdala resting state functional connectivity with the cuneus, occipital lobe (V1), and the fusiform gyrus (altered gating of sensory inputs).  DES scores were positively correlated with left amygdala resting state functional connectivity with the right dlPFC (working memory, attention, inhibition control of emotions). |
| Krause-Utz et al., 2015  Germany | To assess the neural correlates of emotional learning in BPD. | Participants performed an aversive differential delay conditioning paradigm during an fMRI scan. The conditioned stimuli were two neutral graphic images (yellow triangle and blue square) and the unconditioned stimulus was a tolerable electric stimulation of the right thumb.  Skin conductance rates were also collected. | FDS=NR | Participants with BPD showed higher arousal to the conditioned stimulus (CS+) during the extinction phase than healthy controls. Additionally, the BPD group showed CS+ associated amygdala activation whereas the healthy controls did not. No significant correlation with a measure of trait dissociation was observed. |
| Krause-Utz et al., 2018b  Germany | To evaluate brain activity and amygdala functional connectivity in BPD participants with script induced dissociation (BPD-DS) while they are performing an Emotional Working Memory Task (EWMT). | Dissociation was induced in some BPD participants (BPD-DS), while the rest were only exposed to neutral scripts (BPD-NS). All participants completed a EWMT while their brain was imaged using an fMRI machine. | DES:  BPD-DS= 31.74(16.52)  DPD-NS= 26.93(13.50)  HC= 2.68(2.04)  DSS-4  Before Script:  BPD-DS= 3.44(1.99)  DPD-NS= 2.30(1.14)  HC= 1.31(0.66)  After Script:  BPD-DS= 6.85(2.03)  DPD-NS= 1.85(0.84)  HC= 1.19(0.51) | Measures of dissociation were associated with working memory impairments, bilateral amygdala deactivation, and lower activity in the left cuneus, lingual (medial occipito-temporal) gyrus, and the posterior cingulate cortex.  Measures of dissociation were negatively correlated with amygdala functional connectivity with fusiform gyrus, and positively correlated with amygdala functional connectivity with the right middle/superior temporal gyrus and left parietal lobe (emotional salience and working memory/dampening effects). |
| Krause-Utz et al., 2019  Germany | To assess high-frequency heart rate variability in BPD with comorbid PTSD (BPD+PTSD) during resting state and an emotion regulation task. | Participants performed an emotion regulation task where they viewed negative, positive, and neutral images and were asked to either attend to the image or down regulate their emotional reaction using cognitive reappraisal. Electrocardiogram data was collected. | DES:  BPD+PTSD= 31.75(2.38)  BPD= 23.60(1.73)  HC= 5.65(4.09)  DSS-4  Baseline:  BPD+PTSD= 1.55(1.53)  BPD= 1.32(1.51)  HC= 0.00(0.00)  Before Task:  BPD+PTSD= 2.80(2.39)  BPD= 1.66(1.85)  HC= 0.00(0.00)  After Task:  BPD+PTSD= 2.89(2.70)  BPD= 2.07(2.18)  HC= 0.00(0.00) | State dissociation, measured by the DSS-4, in BPD+PTSD was positively correlated with an increase in high-frequency heart rate variability when viewing negative images and asked to down-regulate emotions (F=4.08, p=0.025, R^2^=0.433, R^2^(adjusted)= 0.327). |
| Kremers et al., 2004  Germany | To evaluate autobiographical memory specificity in depressed and non-depressed BPD participants. | Participants completed the Autobiographical memory Test. | DES:  BPD+MDD= 23.4(14.0)  BPD= 21.1(12.1)  MDD=N/A  HC= 9.2(9.7) | BPD+MDD (mean specificity= 59%±19.6) and MDD (mean specificity= 54.4%±25.8) participants had fewer specific memories compared to healthy controls (mean specificity= 75.3%±18.5).  MDD participants had fewer specific memories than BPD participants (mean specificity= 69.8%±18.8).  DES scores were not significantly correlated with specificity of autobiographical memory (r=-0.19). |
| Lange et al., 2005  Germany | To evaluate brain glucose metabolism in temporo-parietal cortices in BPD participants with dissociative symptoms. | Participants were imaged using fluoro-2-deoxyglucouse positron emission tomography (FDG-PET). | DES (subscales)  Absorption:  BPD= 38(15)  HC= 7(5)  Dissociative amnesia:  BPD= 16(16)  HC=2(1)  Depersonalization/derealization:  BPD= 31(17)  HC= 2(2) | DES scores were negatively correlated with resting metabolic rate of the left-sided precuneus and posterior cingulate cortex.  Positively correlated with activation of the right precuneus (interpersonal difficulties). |
| Loffler et al., 2020  Germany | To examine if BPD patients have reduced body ownership. | Participants were interviewed and assessed using the short version of the Survey of Body Area, which divides the body into 25 areas. Participants were asked to relax, close their eyes and rate the percentage (0-100%) of ownership they felt towards different body parts. | FDS= NR | FDS scores were negatively correlated with feelings of body ownership (r = -0.69 p = 0.001). |
| Ludascher et al., 2007  Germany | To assess alterations  in pain and detection thresholds. | Participants experienced a brief pulse of electrical stimulation on their right index finger. They were told to indicate when they perceived the stimulus and when it became painful. | FDS:  BPD= 26.5 (10.41)  HC= 4.8(4.1)  DSS:  BPD= 22.2(14.8)  HC=0.4(0.65) | Higher pain thresholds were observed in BPD participants. DSS and FDS scores were positively correlated with pain threshold (DSS r=0.83, p<0.001; FDS: r=0.80, p<0.01). |
| Ludascher et al., 2009  Germany | To test whether pain perception in BPD patients who currently engage in self harm (BPD+NSSI) is different from patients who had stopped self harm behaviours, and whether pain perception of the latter group differs from healthy controls (HC). | Pain thresholds were assessed using  thermal contact stimuli and laser radiant heat pulses to the participants hand. As temperature or intensity was increased, participants described their perception of the stimulus (nothing, touch, warm, pricking, stinging, burning, and miscellaneous). | FDS:  BPD+NSSI= 24.5(18.9)  BPD= 12.0(7.5)  HC= 3.3(2.7)  DSS:  BPD-NSSI= 0.9(1.1)  BPD= 0.6(0.7)  HC= 0.04(0.1) | DSS and FDS scores were positively correlated with self-harm (DSS: p<0.01; FDS: p<0.001), but not with pain thresholds. |
| Ludascher et al., 2010  Germany | To investigate the psychologic, somatosensory (pain sensitivity) and neural correlates of dissociative states in PTSD and BPD. | Participants brains were imaged using an fMRI machine while being exposed to  a script describing a personalized dissociation-inducing situation and a personalized script describing a neutral situation. | DSS-4  Neutral script:  BPD= 3.4(1.5)  BPD+PTSD= 1.5(0.7)  Dissociation-inducing script:  BPD= 3.0(1.7)  BPD+PTSD= 3.4(1.4) | DSS-4 scores were negatively correlated with pain sensitivity and associated with fronto-limbic activation (p ≤ 0.001). |
| McGirr et al., 2009  Canada | To examine if specific BPD symptoms were more prevalent in persons who died by suicide. | A psychological autopsy was performed on BPD patients who died by suicide. Additionally, a proxy-based interview was conducted with the family and friends of the BPD patient. | SCID-II | Dissociation and paranoid ideation were negatively correlated with suicide (AOR = 0.47, 95% CI 0.25 to 0.89). |
| Merckelbach et al., 2005  Netherlands | To examine the links  among dissociative symptoms, fantasy proneness, and impulsivity in BPD, MDD, and schizophrenia. | Participants completed the following scales: the DES, the CEQ, and the BIS. | DES:  BPD= 31.0 (15.9)  MDD= 17.8(12.8)  SCZ= 21.5(16.5)  DES-T:  BPD= 21.1 (16.8)  MDD= 11.3(14.1)  SCZ= 17.8(18.1) | Measures of trait dissociation were positively correlated with fantasy proneness measured using the CEQ (DES and CEQ: r = 0.55, p<0.05; DES-T and CEQ: r=0.48, p<0.05). |
| Miller et al., 1993  USA | To investigate the impact of substance abuse on patients with BPD. | Participants were assessed using the DSM-III criteria for substance abuse. | DSM-III= NR | Depersonalization-derealization was experienced by 37% of BPD participants with no substance abuse disorder, 28% of those who engaged in alcohol-sedative drug abuse, and 5% of stimulant drug users. The difference between no substance abuse and sedative drug abuse compared to stimulant drug use was significant (x^2^=7.14, df=2, p=0.5). |
| Navarro-Haro et al., 2015  Spain | To investigate the relationship between expressive suppression, cognitive reappraisal, and dissociation with self-harm in BPD patients with an eating disorder (BPD-ED). | Participants underwent clinical interviews and completed the following self-report questionnaires: DES-II and the ERQ. | DES-II= 23.81 (18.7) | Low cognitive reappraisal and increased DES-II scores were positively correlated with increased self-harm in BPD-ED patients. |
| Niedtfeld et al., 2013 Germany | To identify volume abnormalities in limbic regions in BPD, and to evaluate the influence of co-occurring PTSD in BPD patients (BPD+PTSD). | Voxel-based morphometry (VBM) was used to observe gray matter volume (GMV) in participants. | FDS:  BPD + PTSD= 26.7 (15.8)  BPD= 19.5 (8.8) | BPD+PTSD participants had increased GMV in the superior temporal gyrus and the dlPFC. BPD participants had a decrease in GMV in the amygdala and dorsal ACC and right hippocampus.  FDS scores were positively correlated with GMV in middle temporal gyrus (r = 0.34, p < 0.001). |
| Paret et al., 2016  Germany | To assess whether the presence of  emotional stimuli had detrimental effects on operant conditioning  in BPD patients. | An operant conditioning task was performed. Participants underwent four experimental trials where they were either shown an aversive (aversive condition) or neutral (neutral condition) image. During each trial, participants were shown two images and were asked to select the image that was usually rewarded (acquisition and retention phase). Randomly, the image that results in a reward is reversed (reversal phase). | DSS-4  Aversive condition:  BPD= 2.55 (2.50)  HC= NR  Neutral condition:  BPD= 2.26 (2.41)  HC=NR | DSS-4 scores were negatively associated with performance on the acquisition component of the operant conditioning task (higher error rates; r=0.41, p<0.05).  DSS-4 scores and aversive tension were positively correlated (r = 0.56, p<0.01). |
| Pec et al., 2021  Czech Republic | To identify changes in symptoms of patients with BPD enrolled in a psychodynamic day treatment and the factors that predict dropout and clinical outcomes. | Participants were enrolled in a 9-month group psychodynamic day treatment program (open groups of 6-9 participants). Participants were followed-up with a year after the end of treatment. | DES:  Total= 21.71(14.75)  Completers= 20.21(15.17)  Drop-outs= 26.69(11.96) | DES scores were positively correlated with treatment drop out (p=0.046). No significant correlation between DES scores and treatment effectiveness was observed. |
| Popkirov et al., 2019  Germany | To investigate frontal EEG asymmetry (FEA) in BPD. | Participants underwent EEG imaging before and after viewing aversive images (mood induction). Endocrinological effects that resulted from viewing the aversive images were controlled for by measuring salivary cortisol levels. | FDS (Total Score):  BPD= 26.69(16.12)  HC= 6.40(4.23)  FDS (Conversion Score):  BPD= 20.64(16.89)  HC= 2.05(2.72) | BPD participants and healthy controls experienced a left-to-right asymmetry shift in the prefrontal area after mood induction.  The FDS conversion score was positively correlated with leftward frontal EEG asymmetry before mood induction (r =0.667; p <0.001). No difference was observed between the controls and BPD patients after mood induction. |
| Prossin et al., 2010  USA | To investigate the role of the endogenous opioid system and μ-opioid receptors in emotion regulation in BPD. | PET scan was used to measure μ-opioid receptor availability during neutral and sad emotional states. | DES= NR | DES scores were negatively correlated with the nondisplaced binding potential in the right caudate region (r=–0.57, p=0.02). Correlation did not persist after correction for multiple comparisons. |
| Renneberg et al., 2005  Germany | To assess the specificity of autobiographical memory recall in BPD compared to MDD patients. | Participants completed the AMT. This test consisted of five positive, five negative, and five neutral emotional cue words. Participants were asked “What event does this word remind you of?” Latency of their reply and specificity of their answers were measured. | DES:  BPD= 23.9(11.8)  MDD= 18.8(13.5)  HC= 9.5(6.9) | MDD patients had increased latency and fewer specific recalled memories than BPD patients and healthy controls. BPD patients did not differ in recall latency or memory specificity compared to healthy controls. DES scores were not significantly correlated with specificity of recall of autobiographical memory (p>0.31). |
| Rusch et al., 2007  Germany | To evaluate the relationship between inferior frontal region white matter and BPD symptom severity. | Participant’s brains were imaged using Diffusion Tensor Imaging (DTI). | FDS:  BPD= 15.4(0.2)  HC= N/A | FDS scores were positively correlated with mean diffusivity in the right (r= 0.41, p<0.10) and left (r= 0.50, p<0,05) inferior frontal white matter. |
| Russ et al., 1993  USA | To analyse if the presence or absence of pain during self-harm may be a useful criterion for describing subtypes of self-injurious patients with BPD. | Participants completed the following clinical measures: TSC, BDI, HDRS, SPRAS, DES, QED, BDI, lifetime number of suicide attempts, and score for severity of abuse. | DES:  BPD+P= 20.64(15.42)  BPD-NP= 44.23(16.87)  QED:  BPD+P= 13.14(4.07)  BPD-NP= 18.54(5.24) | Measures of trait dissociation were negatively correlated with pain perception during self-harm (p= 0.001). |
| Russ et al., 1996  USA | To use the parameters of sensory decision theory to assess cognitive aspects of pain sensitivity, pain coping strategies, and locus of control orientation as factors that might help explain differences in pain experience between groups of borderline patients. | Participants completed the following clinical measures: SPQ, CSQ, PLOC, DES, SPRAS, and BDI. | DES:  BPD+P= 25.60 (17.00)  BPD-NP= 44.50 (25.30)  HC= 7.82(7.67) | DES scores were negatively correlated with the ability to discriminate between imaginary painful and mildly painful situations (pain insensitive; p < 0.001). |
| Russ et al., 1999  USA | To investigate possible neurophysiological underpinnings of self-injurious  behavior in women with BPD. | Participants were assessed on their self-harm behaviours (pain report). Additionally, they underwent a 4-minute cold pressor test that required participants to immerse their left hand in refrigerated circulating water (10^o^C). EEG recordings were collected during the laboratory pain procedure. | DES:  BPD+P= NR  BPD-NP= NR  MDD= NR  HC=NR | BPD-NP participants experienced less pain during the cold pressor test, and more theta waves than other participants. DES scores were positively correlated with theta activity (r= 0.32, p=0.01) and negatively correlated with pain rating (-0.46, p=0.0001). Pain rating was negatively correlated with theta activity (r=-0.43, p=0.0001). |
| Schmahl et al., 2013  Germany | To examine transcriptional changes associated with states of  tension or dissociation within BPD participants. | Whole blood gene expression profile analysis was conducted using quantitative PCR. For each participant, 2 samples were drawn  during a state of high tension and dissociation, and 2 samples were drawn during non-tension states. | DSS= NR | DSS scores were positively correlated with IL-6 gene expression (p = 0.0119) and negatively correlated with the following genetic markers: GNAS (p = 0.0103), MAPK1 (p = 0.0946), MAPK3 (p = 0.0135), MAPK8 (p = 0.0316), GNAI2 (p = 0.0153), ARRB1 (p = 0.0307), ARRB2 (p = 0.0242), RGS2 (p = 0.0546), IL1B (p = 0.0480), NR3C1 (p = 0.0506), CREB1 (p = 0.0289), PREP (p = 0.0756), SLC18A2 (p = 0.0633). |
| Semiz et al., 2008  Turkey | To determine whether dream anxiety was associated with childhood trauma, dissociative experiences, and subjective sleep  disturbance in BPD participants. | Participants were assessed using the following scales: VDAS, PSQI, DES, and TEC. | DES:  BPD+ND: 53.4(15.8)  BPD-ND: 29.8(7.5)  HC= NR | BPD participants experienced more nightmares, higher levels of dream anxiety, and reduced sleep quality compared to healthy controls. DES scores were positively correlated with dream anxiety (p<0.001). |
| Shearer, 1994  USA | To determine the prevalence of trauma variables and certain behavioral correlates in BPD patients with varying levels of dissociative experience. | Participants were assessed using the following scales: IES, BHS, and Monroe scale for behavioral dyscontrol. | DES= 25.02 (21.19) (range= 0-76) | DES scores were positively associated with behavioural dyscontrol (r=0.57, df=61, p<0.001), hopelessness (r=0.43, df=61, p<0.001), self-harm (t=-4.23, df=60, p<0.0001), alcohol abuse (t=-2.38, df=60, p=0.02), PTSD symptoms (r=0.43, df=48, p<0.001), and more frequent hospital admissions (r=0.41, df=61, p<0.001). |
| Simeon et al., 2003  USA | To investigate the relationship between dissociation and childhood trauma in BPD, and to explore the relationships of dissociation and trauma to various personality features of BPD. | Participants were assessed using the following scales: CTQ-short form, the Tridimensional Personality Questionnaire, the Defense Style Questionnaire, the Relationship Style Questionnaire, and the Schema Questionnaire. | DES:  BPD= 25.80(16.37)  HC= 2.86(3.85) | BPD participants showed higher DES scores and childhood trauma compared with the healthy controls. DES scores did not correlate significantly with childhood trauma (r=0.24, df=18, p=0.31), but were positively correlated with emotional neglect (r=0.48, df=18, p=0.03), primitive defensive structures (r=0.53, df=18, p=0.016), and fearful attachments (r=0.49, df=18, p=0.28). |
| Simeon et al., 2007  USA | To investigate subjective and neurohormonal reactivity to acute psychosocial stress in  BPD as a function of dissociative symptoms. | Participants' basal urinary cortisol and norepinephrine, and plasma cortisol and norepinephrine reactivity to the Trier Social Stress Test (TSST) were assessed. The TSST consists of two parts, a 5-minute preparation phase and a 10-minute presentation phase. The presentation phase consists of a 5-minute job interview speech and 5-minutes of out-loud mental arithmetics. | DES:  BPD-LD= 15.8(8.0)  BPD-HD= 46.4(10.6)  HC= 3.0(2.3) | All three groups did not significantly differ in basal urinary cortisol or norepinephrine levels. The BPD-HD group showed significantly higher peak stress reactivity (r=0.52, p=0.06; higher plasma cortisol levels) than the other two groups. Dissociation, as measured by the DES, may be a marker of higher biological vulnerability to stress. |
| Spinhoven et al., 2006  Netherlands | To investigate whether Autobiographical Memory in BPD improves after TFP or schema focused cognitive behavioral therapy (SF-CBT). | Participants underwent treatment twice a week for 15 months. A modified version of the AMT was conducted. Participants were asked to mention a specific event where they exhibited the word written on the presented card. There were 10 words in total, five negative and five positive words. The AMT was conducted before the start of treatment, nine months after treatment began, and then 15 months after treatment began. | DES:  BPD+MDD= 19.3(14.2)  BPD= 18.6(14.3) | BPD+MDD patients recalled more specific memories and less categorical memories after treatment compared to the BPD only group. DES scores were not significantly correlated with autobiographical memory. |
| Stevens et al, 2004  Germany | To explore the impact of perception speed and working memory in BPD. | Participants underwent a series of auditory and visual delayed matching-to-sample (DMS) tasks. | FDS:  BPD= 15.3(7.2)  HC= 3.3(2.8) | BPD participants required significantly more time to identify visual targets during the backward masking task. Perceptual speed and working memory were impaired in BPD. FDS scores were not correlated with performance on DMS tasks. |
| Wedig et al., 2012  USA | To determine the most clinically relevant baseline and time-varying predictors of suicide attempts in BPD. | Participants were assessed on the following scales for 16 years: DIB-R, DES and DSM-III-R. | DES (measured overtime):  Baseline= 21.8(18.6)  2yr= 15.6(16.5)  4yr= 13(14.9)  6yr= 10.9(13.8)  8yr= 10.4(13.6)  10yr= 8.5(12.1)  12yr= 8.6(12.3)  14yr= 8.7(11.2)  16yr= 8.8(12.2) | DES scores were positively correlated with risk of suicide attempt (OR = 1.02; p=0.002). |
| Wingenfeld et al., 2009  Germany | To assess neural correlates of response inhibition in BPD participants. | Participants were imaged using fMRI while they performed an Emotional Stroop Task (EST) that consisted of neutral, generally negative, and individually negative words. | DSS  Before-EST:  BPD= 0.9(0.9)  HC= 0.2(0.2)  After-EST:  BPD= 1.1(1.1)  HC= 0.1(0.2) | BPD patients had longer reaction times than healthy controls. Healthy controls exhibited increased activation in the ACC and frontal cortex, while BPD patients did not. DSS scores were not significantly correlated with reaction time to BOLD signal changes. |
| Winter et al., 2015  Germany | To examine BPD participants' performance in an emotional Stroop task. | Dissociation was induced through script-driven imagery of a 30-second autobiographical memory in which dissociation was experienced. Other participants were presented with non-emotional scripts. Scripts were read out loud during fMRI by a person unknown to the participant. During the fMRI, an EST that consisted of negative, positive, and neutral words was performed. | DES:  BPD= 28.74(14.30)  BPD+ID= 32.96(16.28)  HC= 3.20 (2.49)  DSS-4  Pre-induction:  BPD= 1.51(1.68)  BPD+ID= 2.08(1.77)  HC= 0.22(4.78)  Post-induction:  BPD= 2.06(2.10)  BPD+ID= 5.75(1.81)  HC= 0.20(0.41) | BPD+ID participants had slower and less accurate responses for negative words versus neutral words compared to the BPD group. Additionally, they had decreased neuronal activity in the fusiform gyrus and parietal cortices independent of word valance, and increased activity in the left inferior frontal gyrus in response to negative words. Induced dissociation was correlated with inefficient cognitive inhibition during EST. |
| Wolf et al., 2011  Germany | To assess functional connectivity of prefrontal and limbic networks in patients with BPD and its association with dissociation. | Resting-state fMRI images of BPD patients were analyzed. | DSS= 38.8 (28.5) | DSS scores were positively correlated with abnormal connectivity in the insula of the default mode network (pain sensitivity; ρ15 = 0.61,  p = 0.009) and negatively correlated with connectivity of the cuneus (ρ15 = –0.58, p = 0.016). |
| Wolf et al., 2012  Germany | To explore the relationship between cerebral blood flow and distinct symptom clusters in BPD. | Participants were imaged using continuous arterial spin labeling magnetic resonance imaging. | DSS:  BPD = 37.3 (28.7)  HC= N/A | BPD participants experienced a decrease in blood flow in the medial orbitofrontal cortex (OFC) and a decrease in left and right lateral OFC, compared to healthy controls. No significant correlation was observed between DSS scores and OFC blood flow. OFC perfusion was positively correlated with impulsivity scores (medial OFC: ρ=0.50, p=0.024; left lateral OFC: ρ=0.58, p=0.009; right lateral: ρ=0.64, p=0.004). |
| Zanarini et al., 2011  USA | To assess the predictors of self-mutilation over 10-years in BPD patients. | Interviews were performed at each follow-up wave (every two years). Participants were assessed on the following scales: the LSDS, the AHI, and the Revised Childhood Experiences Questionnaire (CEQ-R). | DES  2yr= 15.6  4yr= 13  6yr= 10.9  8yr= 10.4  10yr= 8.4 | DES scores were a predictor of self-injurious behaviors overtime (OR= 1.19, SE=0.06, z=3.27, p=0.001). |

*Abbreviations:* AHI, Abuse History Interview; ARRB1, Beta-arrestin-1; ARRB2, Beta-arrestin-2; BDI, Beck Depression Inventory; BDI-II, Beck Depression Inventory version 2; BHS, Becks Hopelessness Scale; BIS, Barratt Impulsiveness Scale; BPD, Borderline Personality Disorder; BPD+D, Borderline Personality Disorder with Dissociation; BPD+ID, Borderline Personality Disorder with Induced Dissociation; BPD+MDD, Borderline Personality Disorder with Comorbid Major Depressive Disorder; BPD+ND, Borderline Personality Disorder with a comorbid nightmare disorder; BPD+NSSI, Borderline Personality Disorder with Non-suicidal Self Injury; BPD+P, Borderline Personality Disorder with Pain during Self-harm; BPD+PD, Borderline Personality Disorder with peritraumatic dissociation; BPD-D, Borderline Personality Disorder without Dissociation; BPD-HD, Borderline Personality Disorder with High Levels of Dissociation; BPD-LD, Borderline Personality Disorder with Low Levels of Dissociation; BPD-ND, Borderline Personality Disorder with a comorbid nightmare disorder; BPD-NP, Borderline Personality Disorder with No Pain during Self-harm; BPD-NSSI, Borderline Personality Disorder without Non-suicidal Self Injury; BPDSI-IV, Borderline Personality Disorder Severity Index- version 4; BSL-95, Borderline Symptom List; cBPD, current Borderline Personality Disorder; CEQ, Creative Experience Questionnaire; CREB1, Cyclic AMP-responsive element-binding protein; CSQ, pain Coping Strategies Questionnaires; CTQ, Childhood Trauma Questionnaire; DES, Dissociative Experience Scale; DES-T, Dissociative Experience Scale- Taxon; dlPFC, Dorsolateral Prefrontal Cortex; DSM-II, Diagnostic and Statistical Manual of Mental Disorders- Second edition; DSM-III, Diagnostic and Statistical Manual of Mental Disorders- Third Edition; DSM-III-R, Diagnostic and Statistical Manual of Mental Disorders- Third Edition Revised Version; DSS, Dissociative Tension Scale; DSS-4, Dissociative Tension Scale- Short Version; EEG, Electroencephalogram; EMG, Electromyography; ERQ, Emotion Regulation Questionnaire; FDS, Fragebogen zu Dissoziativen Symptomen; FFMQ, Five Facets Mindfulness Questionnaire; fMRI, Functional Magnetic Resonance Imagining; GHQ, General Health Questionnaire; GNAI2, Guanine nucleotide-binding protein G(i) subunit alpha-2; GNAS, Guanine nucleotide-binding protein G(s) subunit alpha isoforms; HC, Healthy Control; HDRS, Hamilton Depression Rating Scale; HPA-Axis, Hypothalamic Pituitary Adrenal Axis; IES, Impact of Event Scale; IL1B, Interleukin-1B; IL-6, Interleukin-6; LSDS, Lifetime Self-Destructiveness Scale; MAPK1, Mitogen-activated protein kinases 1; MAPK3, Mitogen-activated protein kinases 3; MAPK8, Mitogen-activated protein kinases 8; MDD, Major Depressive Disorder; MRI, Magnetic Resonance Imagining; MRS, Magnetic Resonance Spectroscopy; NR, Not Reported; NR3C1, Glucocorticoid receptor; OCD, Obsessive Compulsive Disorder; PCR, Polymerase Chain Reaction; PET, Positron Emission Tomography; PLOC, Pain Locus of Control; PREP, Prolylen-dopeptidase; PSQI, Pittsburgh Sleep Quality Index; PTSD, Posttraumatic Stress Disorder; QED, Questionnaire for Experiences of Dissociation; QNSSI, Questionnaire for Non-Suicidal Self-Injury; rBPD, remitted Borderline Personality Disorder; RGS2, Regulator of G-protein signaling 2; SCID-D, Structured Clinical Interview for the DSM- Dissociative Disorders; SCID-II, Structured Clinical Interview for the DSM- Axis two disorders; SCL-90-R, Symptom Checklist; SCZ, Schizophrenia; SEQ, Sexual Experience Questionnaire; SLC18A2, Synaptic vesicular amine transporter; SMQ, Schema Mode Questionnaire; SPQ, Situational Pain Questionnaire; SPRAS, Sheeha Patient Rated Anxiety Scale; TEC, Traumatic Experience Checklist; TSC, Trauma Symptom Checklist; V1, Primary Visual Cortex; WDS, Wessex Dissociation Scale; Yr, year.
